# Bovine viral diarrhea virus (BVDV) relies on cellular lipid droplet biogenesis and lipolysis to provide energy for viral replication

**DOI:** 10.1186/s13567-026-01834-7

**Published:** 2026-09-19

**Authors:** Xiaoran Xiong, Yi Liu, Yaohong Zhu, Jiufeng Wang, Guiyan Yang

**Affiliations:** State Key Laboratory of Veterinary Public Health and Safety, College of Veterinary Medicine, China Agricultural University, No. 2 Yuanmingyuan West Road, Beijing, 100193 People’s Republic of China

**Keywords:** Bovine viral diarrhea virus, core protein, lipolysis, mitochondria, fatty acid oxidation, viral replication

## Abstract

Bovine viral diarrhea virus (BVDV), one of the most important viral pathogens in cattle, causes serious economic losses due to immunosuppression and persistent infections. Previous studies have shown a tight connection between virus infection and lipid metabolism, particularly in the formation and degradation of lipid droplets (LDs). However, the pathogenic mechanism of BVDV infection and the molecular mechanisms by which viral proteins reprogram lipid metabolism remain unclear. We found that BVDV increased cellular LD accumulation by enhancing the production of new lipids, accelerating exogenous fatty acid uptake, and elevating diacylglycerol acyltransferase (DGAT)-dependent esterification of fatty acids (FAs). The generated LDs subsequently release free fatty acids (FFAs) via adipose triglyceride lipase/hormone-sensitive lipase (ATGL/HSL)-dependent lipolysis. This lipolysis-dependent release of FFAs is accelerated and transferred to mitochondria for oxidation by increasing contact between LDs and mitochondria, thereby promoting viral replication. Furthermore, BVDV core protein targets the surface of LDs, increasing and recruiting fatty acid synthase (FASN) and ATGL to promote LD mobilization. Meanwhile, the core protein interacts with mitochondria, linking mitochondria to LDs and facilitating the release of FAs for efficient fatty acid oxidation. Collectively, this study demonstrates that the BVDV core protein regulates cellular lipid metabolism to support BVDV replication, contributing to understanding the pathogenetic mechanisms by which BVDV interacts with host cells.

**Supplementary Information:**

The online version contains supplementary material available at https://doi.org/10.1186/s13567-026-01834-7.

## Introduction

Bovine viral diarrhea virus (BVDV) is one of the most important viral pathogens of cattle worldwide. It is an enveloped, single-stranded, positive-sense RNA virus belonging to the genus *Pestivirus* within the family *Flaviviridae*, which encodes a large polyprotein that undergoes proteolytic cleavage to form the structural proteins core, Erns, E1, and E2 and nonstructural proteins Npro, P7, NS2, NS3, NS4A, NS4B, NS5A, and NS5B [1]. Infection with BVDV can cause diverse clinical manifestations in cattle, including respiratory disease, immunosuppression, reproductive disorders, persistent infection, and mucosal disease characterized by severe mucosal necrosis [2]. The prevalence of BVDV infection in different geographic regions ranges from 28% to 66% in cattle herds, with 0.5–2.5% of livestock persistently infected (PI) with the virus [2]. Due to immunosuppression and high genomic variability, which enable rapid adaptation and persistent infection within herds, the emergence of new subtypes and reduced efficiency of vaccines lead to severe economic losses [3]. BVDV relies on host cellular factors and machinery to complete its life cycle, involving complex interactions with cellular lipid metabolism [4]. Despite their role as dynamic hubs of lipid and energy homeostasis, the involvement of lipid droplets (LDs) in BVDV infection remains undefined.

LDs are cellular organelles composed of a neutral lipid core and surrounded by a phospholipid monolayer decorated with a specific set of proteins [5]. LD biogenesis begins in the endoplasmic reticulum (ER), where the diacylglycerol acyltransferase (DGATs) enzymes esterify fatty acids (FAs) from either de novo lipogenesis (DNL) or exogenous uptake into triacylglycerol (TAG) deposited between the leaflets of the ER bilayer [5]. LDs can be mobilized by releasing free fatty acids (FFAs) via lipophagy and lipolysis [6]. In lipophagy, LDs are delivered to autolysosomes or lysosomes for degradation by lysosomal acid lipase (LAL) [6]. In lipolysis, cytosolic neutral lipases bind to the surface of the LDs to hydrolyze triacylglycerols [6].

Viral infections often correlate with accelerated LD biogenesis or catabolism. FAs derived from LDs comprise the cellular FAs pool that performs several functions and is co-opted by diverse viruses for various purposes: (i) producing phospholipids for viral replication organelles (ROs); (ii) modifying viral proteins by palmitoylation; and (iii) re-esterifying to LDs for infectious virus assembly or providing substrates for fatty acid oxidation (FAO) to produce ATP [7]. These studies indicate that lipid metabolic reprogramming and LD formation may play crucial roles in the pathophysiology and replication of BVDV.

LDs are in contact with many organelles and provide the necessary lipids for biogenesis [5]. Beta-oxidation of FAs in mitochondria dominates the process of energy production [8]. Cellular FAs are activated into acyl-CoA and transported to the mitochondria via the carnitine cycle. Within the mitochondria, they are then progressively degraded to produce energy through a series of cyclical reactions that include dehydrogenation, hydration, another dehydrogenation, and finally thiolytic cleavage [8]. LDs can dynamically associate with subpopulations of mitochondria in different cell types or under different metabolic conditions [9]. Contacts between LDs and mitochondria could serve as sites for both lipogenesis and lipolysis. However, whether BVDV infection regulates mitochondrial function for β-oxidation and the contact between LDs and mitochondria remains largely unknown.

LDs and mitochondria have a recognized involvement in the life cycle of *Flaviviridae* family viruses. The capsid proteins of hepatitis C virus, dengue virus, and Zika virus from the *Flaviviridae* family bind to the LDs via relatively conserved residues. These viruses usurp the LDs and alter the lipidomic profiles and LD-associated innate immunity to enhance their viral processes [10–12]. Other viral proteins from the *Flaviviridae* family have been shown to localize to mitochondria, alter host mitochondrial dynamics and β-oxidation function, and interfere with mitochondrial antiviral signaling to promote viral replication [13, 14]. However, it remains unclear whether cellular LDs and mitochondrial homeostasis are impacted by BVDV infection, which viral proteins are involved in the infection process, and what the importance of these processes is for understanding the pathophysiology of BVDV infection.

This study reveals that BVDV infection promotes intracellular accumulation of LDs, which are subsequently mobilized by the virus for lipolysis. By increasing contact between LDs and mitochondria, lipolysis-dependent FFAs are accelerated and transferred to mitochondria for oxidation, thereby promoting viral replication. We also found that BVDV core protein targets the surface of LDs, increasing and recruiting fatty acid synthase (FASN) and adipose triglyceride lipase (ATGL) to promote LD mobilization. At the same time, core proteins interact with mitochondria, linking mitochondria to LDs and providing an opportunity to release FAs for more efficient FAO. Our findings contribute to elucidating the pathogenetic mechanism of BVDV infection and exploring targets for medical products to control BVDV transmission.

## Materials and methods

### Cell culture and virus infection

Madin–Darby bovine kidney (MDBK) cells and bovine endometrial epithelial cells (BEECs) were cultured in Dulbecco’s modified Eagle’s medium (DMEM) (SH30243.01, Cytiva) supplemented with 1% antibiotic–antimycotic (15,140,163, Gibco) and 10% fetal bovine serum (FBS) (12303C, Sigma-Aldrich). The cells used in the study tested negative for *Mycoplasma* spp. The BVDV AV67 strain was obtained from the China Veterinary Culture Collection (Beijing, China) and stored in our laboratory for use in subsequent experiments. MDBK cells and BEECs were mock-infected or infected with BVDV strain AV67 at a multiplicity of infection (MOI) of 0.1, 1, and 5. After 1 h of absorption at 37 °C in a 5% CO_2_ incubator, the cells were washed three times with PBS to remove unbound virus and then cultured at 37 °C in DMEM supplemented with 2% FBS.

### Antibodies and reagents

Antibodies against green fluorescent protein (GFP) (mouse, HRP-66002), P62 (rabbit, 18420-1-AP), ATGL (rabbit, 55190-1-AP), HSL (rabbit, 17333-1-AP), LAL (rabbit, 12956-1-AP), LAMP1 (rabbit, 21997-1-AP), TOMM20 (rabbit, 11802-1-AP), FASN (rabbit, 10624-2-AP), and β-actin (mouse, 66009-1-Ig) were purchased from Proteintech. Antibodies against LC3 (rabbit, #12741) was obtained from Cell Signaling. Antibody against dsRNA (J2) was obtained from Cell Signaling. Horseradish peroxidase (HRP)-conjugated goat anti-mouse or rabbit immunoglobulin (Ig)G were obtained from Proteintech. Alexa Fluor 488/568-conjugated goat anti-mouse or rabbit IgG were purchased from Life Technologies. Anti-eGFP magnetic agarose beads (HY-K0246), palmitic acid (HY-N0830), and Nile Red (HY-D0718) were obtained from MCE. BODIPY 493/503 (D3922) and BODIPY 558/568 C12 (D3835) were purchased from ThermoFisher Scientific. Oleic acid (O1008) and the DGAT2 inhibitor (PF-006424439) were purchased from Sigma-Aldrich. BODIPY 500/510 (C2055) was purchased from Beyotime. Orlistat (HY-B0218), Torin 1 (HY-13003), PF-04620110 (HY-13009), Bafilomycin A1 (HY-100558), Atglistatin (HY-15859), CAY10499 (HY-119283), 2-BP (HY-111770), and Etomoxir (HY-50202) were obtained from MCE. 5-aminoimidazole-4-carboxamide ribonucleoside (AICAR) (S1802) was purchased from Selleck.

### Cell viability assay

Cell viability was determined using a Cell Counting Kit-8 (C0038, Beyotime). In brief, 5000 cells per well were seeded in a 96-well plate and allowed to grow in the medium overnight. On the second day, the medium was removed, and the cells were incubated with a given concentration of the compound for 36 h. At the end of each treatment, the medium was replaced, and CCK-8 (10 mL) was added to each well and incubated at 37 °C for 2 h. After the cells were incubated for another 2 h, the absorbance was detected at 450 nm. The effects of the compounds on cell viability are shown in Additional file 2.

### Quantitative real-time PCR

Total RNA was extracted from mock or BVDV-infected MDBK cells using a total RNA kit (R6834-01, Omega), and cDNA was synthesized using a TransStart one-step gDNA removal and cDNA synthesis supermix kit (AE341-02, TransGen) according to the manufacturer’s instructions. Glyceraldehyde-3-phosphate dehydrogenase (GAPDH) and β-actin were used as reference genes. Results are presented as fold change compared with matched controls using the 2^−ΔΔCT^ method. Primer sequences are presented in Table 1.

**Table 1:** **Primers used in this study**

Gene	Direction	Sequence (5'-3')
*PLIN2*	F	AGTGAACTTGCCAGGAAGAATG
	R	TTCATCTGTATCATCGTAGCCG
*ACLY*	F	GTCAACCTCACTCTGGATGGA
	R	TCGTGGTGGAACAGGACATAG
*ACC1*	F	GGGTGAAAGACTGGGTTGAA
	R	GACAGAGCACGGATGTGATG
*FASN*	F	TCCCACTGGAACAAGACAAGC
	R	TTTCTTTTGAAGTTGAGGGAGGC
*SCD1*	F	TCCGACCTAAGAGCCGAGAA
	R	TGGGCAGCACTATTCACCAG
*CD36*	F	GGTCCTTACACATACAGAGTTCG
	R	ATAGCGAGGGTTCAAAGATGG
*FATP*	F	GGGGCAGTGTCTCATCTATGG
	R	CCGATGTACTGAACCACCGT
*FABP4*	F	TGGGATGGAAAATCAACCAC
	R	TGGCTTATGCTCTCTCATAAAC
*DGAT1*	F	CCACTGGGACCTGAGGTGTC
	R	GCATCACCACACACCAATTCA
*DGAT2*	F	AATCGCAAGGGCTTTGTGAAAC
	R	CCCCAGGTGTCAGAGGAGAAGA
*ATGL*	F	CTCTGATGGCGAGAACGTCA
	R	TTGTCTGAGATGCCACCGTC
*HSL*	F	AGTCCCACCTGAAATCAGTGT
	R	CCAAGTAAGAAGTTGATGGTT
*MGL*	F	GCAACCAGCTGCTCAACAC
	R	AGCGTCTTGTCCTGGCTCTT
*PPAR-A*	F	CGGTGTCCACGCATGTGA
	R	TCAGCCGAATCGTTCTCCTAAA
*PGC1-A*	F	GTGAAGACCAGCCTCTTTGC
	R	TCACTGCACCACTTGAGTCC
*TFAM*	F	GGCACATCACAGGTAAAGC
	R	AATTGAGCGACGTATAAGAT
*CPT1A*	F	CAAAACCATGTTGTACAGCTTCCA
	R	GCTTCCTTCATCAGAGGCTTCA
*CPT2*	F	TGCCTTCCTTCCTGTCTTGGTATG
	R	GGGTCTGGGTAAACGAGTTGAATTG
*VLCAD*	F	TTCCATACCCGTCTGTGCTCAAC
	R	GGCAGCATCGTTCACCTCCTC
*MCAD*	F	CAGACACCCCAGGAGTACAGATTG
	R	TTCCTTGGGCACTCTCACATCTTC
*SCAD*	F	CTGGACTGTGCTGTGACCTACG
	R	GCCATGTCCGCCAACTTGAAC
*BVDV-5'UTR*	F	TAGTCGTCAGTGGTTCGACGC
	R	CCTCTGCAGCACCCTATCAG
*PLIN3*	F	GAGCGGGGTGGACACAGTGC
	R	CAAGGGATGTGGCGAGGCGG
*β-actin*	F	AAGGACCTCTACGCCAACACG
	R	TTTGCGGTGGACGATGGAG

### Virus titration

The MDBK cells were incubated with BVDV at 37 °C for 1 h, after which the unbound viruses were washed off and the cells were cultured in supplemented medium. After repeated freezing and thawing twice, the whole-cell culture supernatant was harvested by centrifugation. This was then serially diluted tenfold to infect MDBK cells in 96-well plates. After incubation at 37 °C for 24 h, the cells were fixed with 4% paraformaldehyde for 10 min at room temperature, and an immunofluorescence assay was performed using a mouse anti-dsRNA (J2) antibody and an Alexa Fluor 488-labeled goat anti-mouse IgG secondary antibody to visualize viral infection. Viral titers were measured using the Reed–Muench assay and expressed as the logarithm of the infectious dose per 0.1 mL of 50% tissue culture infective dose (TCID_50_/0.1 mL).

### Western blotting

Cells were lysed on ice for 20 min in radioimmunoprecipitation assay (RIPA) buffer (R0010, Solarbio) supplemented with proteases/phosphatase inhibitor cocktail (#5872, Cell Signaling). Protein samples were subjected to electrophoresis on 8%, 10%, or 15% gradient SDS-PAGE gels, depending on the molecular weight of the protein, and then transferred to a polyvinylidene fluoride membrane (HATF00010, Millipore). After blocking in 5% skimmed milk for 1.5 h at room temperature, membranes were washed three times for 5 min each with PBST and incubated with appropriate primary antibodies overnight at 4 °C, followed by HRP-conjugated secondary antibodies for 1.5 h at room temperature. Targeted protein bands were detected using an enhanced chemiluminescence (ECL)-enhanced chemiluminescence detection system (Tanon 6200, Shanghai, China). The bands were quantified by densitometry using Fiji software (version 1.50).

### Confocal fluorescence microscopy

For immunostaining, cells grown on climbing sheets were washed three times with PBS, fixed with 4% paraformaldehyde for 15 min, and permeabilized with 0.1% Triton X-100 in PBS for 15 min. Cells were blocked with 2% bovine serum albumin in PBST for 60 min. Primary antibodies were incubated in 1% BSA overnight at 4 °C, followed by Alexa Fluor-conjugated secondary antibody for 1 h at room temperature. LDs were stained with BODIPY 493/503, BODIPY 558/568 C12, or Nile Red in the dark for 30 min, and nuclei were stained with 4,6-diamidino-2-phenylindole (10236r,276001, Sigma-Aldrich) for 5 min at room temperature. The coverslips were then mounted onto slides and visualized using a Nikon A1 confocal laser scanning microscope.

### Fluorescent fatty acid uptake

For the fatty acid uptake assay, MDBK cells were either mock-infected or BVDV-infected at a MOI of 5. After 1 h of absorption, cells were washed three times with PBS and maintained in fresh phenol red-free DMEM. At 11.5 hpi, cells were washed with PBS and incubated for 30 min at room temperature in the dark in DMEM medium containing 5 μM of the fluorescent fatty acid analogue BODIPY 500/510. At 12 hpi, the cells were fixed with 4% PFA. BODIPY 500/510 uptake was quantified by measuring the intracellular green fluorescence signal by confocal imaging.

### Fluorescence fatty acid labeling

The transfer of lipids from LDs to mitochondria was monitored as previously described [9]. In brief, BODIPY 558/568 C12 was added to the culture medium of MDBK cells at a final concentration of 2 μM for 8 h before infection, followed by three washes with PBS and virus infection at MOI = 3 for 1 h. After 1 h of absorption, cells were then washed and chased in DMEM supplemented with 1% FBS for 12 h and then fixed with 4% PFA. TOMM20 antibody was used as a mitochondrial marker for cell staining and visualization by confocal imaging.

### Fluorescent image analysis

The fluorescent images used for phenotype quantification were acquired below saturation using identical parameters (i.e., photomultiplier tube high voltage, Photomultiplier tube offset, laser power, and magnification) and processed using Fiji software. The Analyze Particles macro was used to measure the number and size of LDs in thresholded images, and the Pearson’s correlation coefficient and Manders’ colocalization coefficient in two channels were calculated to quantify colocalization using the JACoP plugin in Fiji software. The two-dimensional (2D) traces of the tubular mitochondrial network were obtained by the Skeletonize 2D/3D plugin of ImageJ. Brightness and contrast were adjusted using identical settings in Adobe Photoshop, and panels were assembled using Adobe Illustrator. The images shown are representative of at least three independent experiments.

### Cellular triacylglycerol, total cholesterol, and free fatty acid measurement

Cells treated in different experiments were harvested and washed three times with PBS. Intracellular triacylglycerol, cholesterol, and free fatty acids were measured using the triglyceride assay kit (BC0620, Solarbio), total cholesterol assay kit (BC1980, Solarbio), and Amplex Red Free Fatty Acid Assay Kit (BC0590, Solarbio) according to the manufacturer’s protocols. Results were standardized to the total cellular protein content.

### Cellular ATP measurement

ATP was measured using an ATP assay kit (S0026, Beyotime) according to the manufacturer’s protocols. Results were standardized to the total cellular protein content.

### DNA construction and transfection

The plasmids encoding C-terminus flag tagged BVDV strain AV67 core proteins were generous gifts from Jing Wang (Zhejiang A&F University). The pEGFP-C1 and pEGFP-N1 vectors were stored in our laboratory. Core-FLAG was used as a template to generate the N-terminus tagged mammalian expression plasmids p-EGFP-Core and the C-terminus tagged expression plasmid p-Core-EGFP. Plasmid DNAs were transfected into bovine endometrial epithelial cells using Lipofectamine 3000 (L3000015, ThermoFisher) according to the manufacturer’s instructions. Transfection efficiency was detected by immunofluorescence of the EGFP tag.

### Immunoprecipitation

Bovine cells were transfected with the pEGFP-C1 or pEGFP-Core plasmid and cultured at 37 °C in 5% CO_2_ for 36 h. Next, the cells were collected and lysed in IP lysis buffer (P0013, Beyotime) containing protease inhibitor phenylmethylsulfonyl fluoride (PMSF) (P8340, Solarbio). Lysates were centrifuged at 12,000 g for 15 min at 4 °C; 20% of the supernatant was saved as input for immunoblotting analysis. Meanwhile, 20 μL of anti-eGFP magnetic agarose beads (HY-K0246, MCE) were washed with lysis buffer, added to the remaining protein samples, and incubated overnight at 4 °C. Subsequently, the beads were washed three times with PBS and eluted with 2 × SDS loading buffer. Co-immunoprecipitated proteins were analyzed by western blot.

### Statistical analysis

All statistical analyses were performed with GraphPad Prism V9.0 software. For quantitative analyses, data from at least three independent experiments are expressed as means ± SEM to ensure reproducibility. Data were analyzed using a two-tailed Student’s *t* test or a one-way analysis of variance (ANOVA; ns, *p* > 0.05; *, *p* < 0.05; **, *p* < 0.01; ***, *p* < 0.001). No data were excluded from the analyses.

## Results

### BVDV upregulates LD biogenesis in favor of viral replication

**Figure 1:**
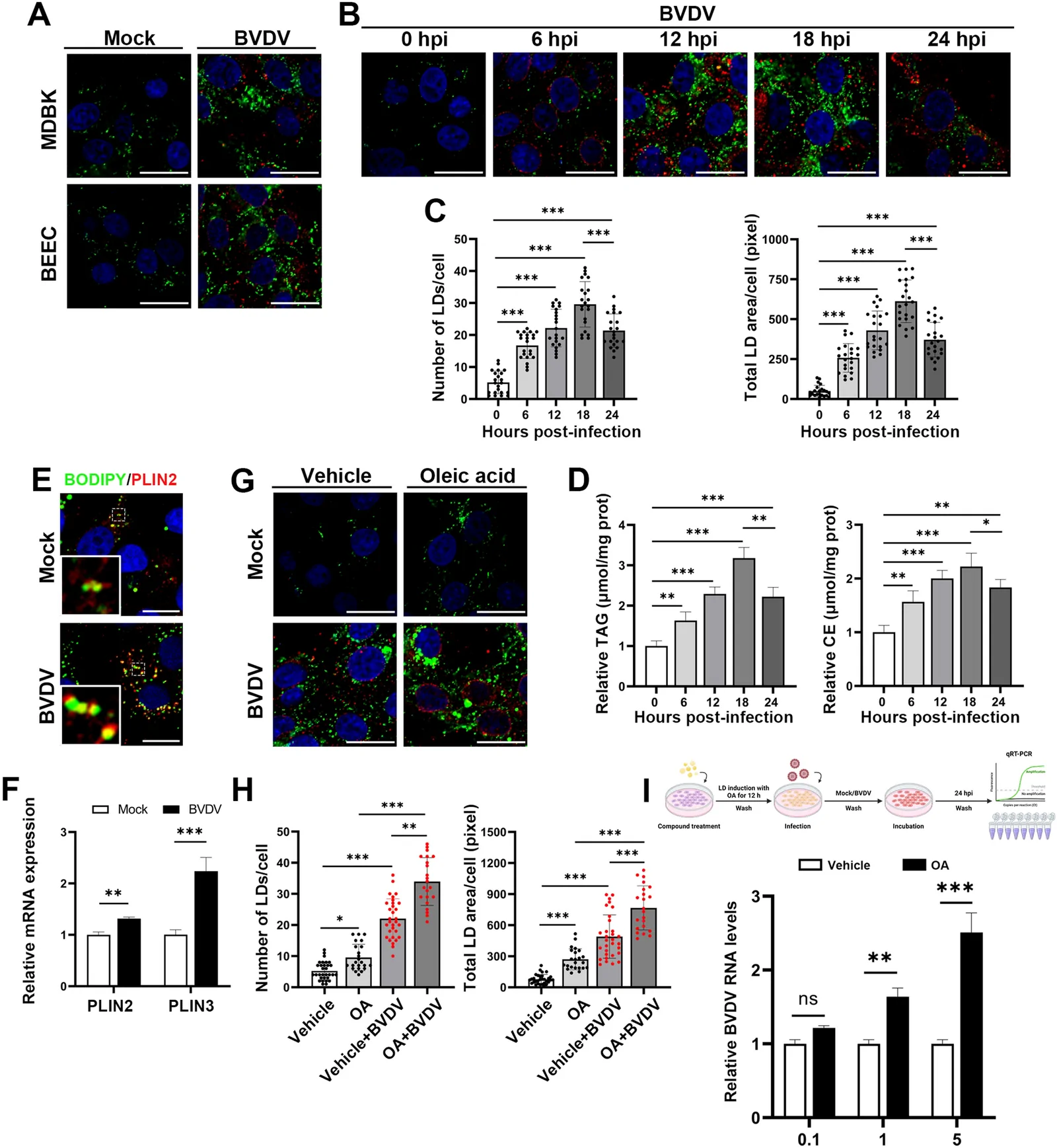
**BVDV upregulates LD biogenesis in favor of viral replication.** **A** MDBK cells and BEECs were infected with BVDV strain AV67 at a MOI of 5 and fixed at 12 hpi. All cells were stained with BODIPY 493/503 to visualize LDs (green) and DAPI to visualize the cell nuclei (blue). BVDV was detected using the anti-dsRNA (J2) antibody (red). Images are representative of three independent experiments. The scale bar represents 20 μm. **B** MDBK cells were treated as in panel A and fixed at the indicated timepoints. **C** MDBK cells were treated as in panel B. The average number of LDs per cell and total LD area per cell were quantified using the Analyze Particles function of ImageJ (> 20 cells; *n* = 3). **D** MDBK cells were treated as in panel B. Cellular lipids were extracted, and the amounts of TAGs and TC were quantified. **E** Mock or BVDV-infected (MOI = 5) MDBK cells were incubated in DMEM. At 12 hpi, cells were fixed and stained with anti-perilipin 2 (red) and BODIPY 493/503. **F** MDBK cells were treated as in panel E. The mRNA expression of *PLIN2* and *PLIN3* was quantified. **G** MDBK cells were treated with 200 μM oleic acid (OA) for 12 h. **H** MDBK cells were treated as in panel G. The average number of LDs per cell and total LD area per cell were also quantified (> 20 cells; *n* = 3). **I** Schematic representation of lipid loading from OA treatment to promote BVDV replication. MDBK cells were pretreated with or without 200 μM OA for 12 h and then infected with BVDV at different MOIs (0.1, 1, and 5). BVDV replication was assessed by qRT-PCR at 24 hpi. Data are means ± SEM. Statistical significance was determined using unpaired Student’s *t* test or one-way ANOVA (ns, *p* > 0.05; *, *p* < 0.05; **, *p* < 0.01; ***, *p* < 0.001).

To investigate the role of lipid droplets (LDs) in the BVDV life cycle, we examined the abundance and morphology of these organelles in BVDV-infected bovine cells by immunofluorescence analysis. In this study, BODIPY 493/503, a hydrophobic dye, was used to visualize LDs [15]. We also used an anti-dsRNA antibody to detect BVDV infection. At 12 h post-infection (hpi), BVDV caused an upregulation of LDs in Madin–Darby bovine kidney (MDBK) cells and bovine endometrial epithelial cells (BEECs) (Figure 1A). To further define the dynamics of LD induction following BVDV infection, we set up a time-course analysis. BODIPY staining revealed significant changes in both number and area of LDs in a time-dependent manner during BVDV infection in MDBK cells (Figure 1B–C). Lipid content analysis showed that LDs and their major constituents, triacylglycerols (TAGs) and total cholesterol (TC), were continuously elevated up to 18 hpi but decreased at 24 hpi (Figure 1D). In addition, perilipin 2 (PLIN2), a well-known surface marker that maintains LD stability [16], was upregulated and localized around LDs displaying typical doughnut-like circular structures upon infection (Figure 1E). The mRNA levels of PLIN2 and PLIN3 were also significantly upregulated by BVDV (Figure 1F). Furthermore, we tested whether MDBK cells and BEECs treated with oleic acid (OA) could further induce LDs. As expected, the number and area of LDs were increased in both MDBK cells (Figure 1G-H) and BEECs (Additional file 1) supplemented with 200 μM oleic acid for 12 h compared with the BSA vehicle. In the early stage of the BVDV life cycle (12 hpi), BVDV was able to further upregulate cellular LD levels in OA-treated bovine cells (Figure 1G-H, Additional file 1).

LDs may exert antiviral or proviral effects at different stages of viral infection [17]. We therefore investigated the role of LDs in the BVDV life cycle. MDBK cells were pretreated with 200 μM OA for 12 h, followed by infection with BVDV at a multiplicity of infection (MOI) of 0.1, 1, and 5. At 24 hpi, quantitative reverse transcription polymerase chain reaction (qRT-PCR) results showed that intracellular BVDV RNA levels were increased by OA except for MOI = 0.1 (Figure 1I). This increase in viral load suggests that enhanced LD accumulation exerts a proviral replication effect. Together, these data indicate that BVDV modulates cellular lipid homeostasis and LD formation throughout its life cycle, and that increased cellular LDs promote BVDV replication.

### BVDV promotes LD biogenesis by stimulating de novo lipogenesis

**Figure 2:**
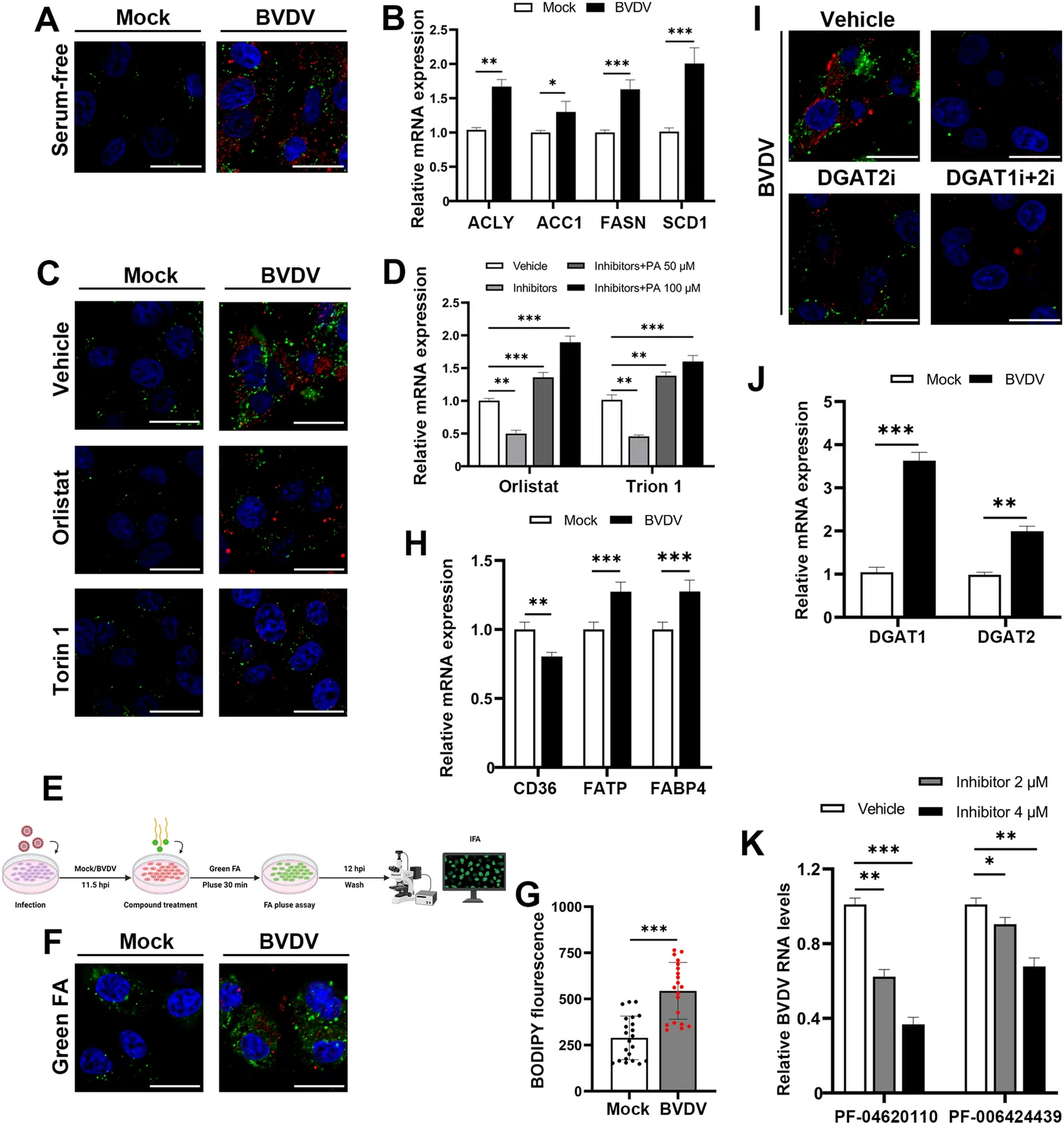
**BVDV increases the number of cellular LDs by promoting de novo lipogenesis, exogenous fatty acid uptake, and DGAT-dependent FA esterification.** **A–B** Mock or BVDV-infected (MOI = 5) MDBK cells were incubated in serum-free maintenance medium for exogenous fatty acid uptake. At 12 hpi, cells were fixed and stained with BODIPY 493/503 for LDs (green) and DAPI for cell nuclei (blue). BVDV was detected using the anti-dsRNA (J2) antibody (red). **B**, **H**, **J** qRT-PCR analysis of DNL, FA uptake, and FA esterification genes at 12 hpi (MOI = 5). **C** Mock or BVDV-infected (MOI = 5) MDBK cells were incubated at 1 hpi with DMSO, 5 μM Orlistat, or 500 nM Torin 1, respectively. At 12 hpi, cells were fixed and stained with BODIPY 493/503 for LDs (green) and DAPI for cell nuclei (blue). **D** BVDV-infected (MOI = 1) MDBK cells were treated with Orlistat, Torin 1, and palmitate for 24 h. Intracellular BVDV RNA was detected at 24 hpi. **E** Schematic of the fatty acid uptake assay. **F–G** Mock or BVDV-infected (MOI = 5) MDBK cells were maintained in fresh phenol red-free DMEM. At 11.5 hpi, cells were incubated for 30 min in DMEM containing 5 μM BODIPY 500/510. At 12 hpi, cells were fixed and stained with DAPI for cell nuclei and fluorescence intensity of BODIPY 500/510. **H–I** Mock or BVDV-infected (MOI = 5) MDBK cells were incubated in a maintenance medium. BVDV-infected cells were incubated at 1 hpi with DMSO, 4 μM PF-04620110, 4 μM PF-006424439, or combined PF-04620110 and PF-006424439, respectively. At 12 hpi, cells were fixed and stained with BODIPY 493/503 for LDs and DAPI for cell nuclei. **K** BVDV-infected (MOI = 1) cells were treated with PF-04620110 or PF-006424439 for 24 h. Intracellular BVDV RNA was quantified at 24 hpi. Data are means ± SEM. Statistical significance was determined using unpaired Student’s *t* test or one-way ANOVA (ns, *p* > 0.05; *, *p* < 0.05; **, *p* < 0.01; ***, *p* < 0.001).

We then investigated the mechanism by which BVDV infection causes intracellular LD accumulation. In a serum-free maintenance medium, BVDV induced LD formation (Figure 2A), suggesting that virally driven LD induction is associated with de novo lipogenesis (DNL). Consistently, the mRNA levels of lipogenic genes *Acly, Acc1, Fasn,* and *Scd1* were upregulated by BVDV (Figure 2B).

To further investigate the contribution of DNL to LD biogenesis, we assessed LDs by confocal microscopy at 12 hpi using two pharmacological inhibitors: Orlistat, to inhibit the activity of the DNL rate-limiting enzyme FASN [18], and Torin 1, to inhibit mechanistic target of rapamycin complex 1 (mTORC1) activity and subsequent sterol regulatory element-binding protein 1c (SREBP-1c) cleavage [19]. Both inhibitors significantly reduced BVDV-induced LDs to basal levels without affecting cell viability, demonstrating that BVDV-mediated LD biogenesis relies on the DNL pathway (Figure 2C). qRT-PCR results showed that Orlistat or Torin 1 treatment inhibited BVDV replication in a dose-dependent manner at 24 hpi (Figure 2D). Inhibition of FASN eliminates the production of end-product palmitate and leads to aggregation of earlier pathway intermediates malonyl-CoA [20]. To validate the specificity of these inhibitors, we investigated whether adding exogenous palmitate would restore BVDV replication. Compared with the BSA vehicle, adding BSA-conjugated palmitic acid (PA-BSA) fully reversed the antiviral effects of Orlistat in a dose-dependent manner (Figure 2D). Taken together, these results indicate that FASN-mediated lipogenesis is important for LD formation and BVDV replication.

### BVDV increases exogenous fatty acid uptake

In addition to enhancing de novo FA synthesis, viruses also require the uptake of FAs [21]. Given that PA-BSA fully restores BVDV replication in the FASN-inhibited state, fluorescent FA uptake experiments using the green fluorescent FA analogue BODIPY 500/510 were performed to determine the contribution of exogenous lipids to increasing cellular lipid content [22]. In this paradigm, mock or BVDV-infected MDBK cells were maintained in serum-free medium, making the green FAs the only exogenous FAs available. MDBK cells were incubated with BODIPY 500/510 for 30 min, and then uptake was quantified (Figure 2E). Immunofluorescence analysis of BVDV-infected cells revealed that BODIPY 500/510 aggregated in the cytoplasm into rounded bright structures, and the total fluorescence intensity of intracellular green FAs was significantly increased compared with the mock (Figure 2F–G). Fatty acid uptake and transport require a series of enzymes such as FATP, FABP4, and CD36 [23]. Our results revealed that *FATP* and *FABP4* mRNA levels were significantly increased in BVDV-infected cells, while *CD36* mRNA was significantly reduced compared with mock-infected cells (Figure 2H).

### DGAT is necessary for LD biogenesis during BVDV infection

Diacylglycerol acyltransferase (DGAT) enzymes are responsible for the final step in TAG synthesis, esterifying diacylglycerol (DAG) to TAG, which is then packaged into LDs. Esterification mediated by DGATs is utilized by many viruses to promote viral replication [24, 25]. To further investigate the role of these two enzyme isozymes on LD formation during BVDV infection, virus-infected cells were treated with DGAT pharmacological inhibitors (DGATi). At 12 hpi, MDBK cells treated with DGAT1 inhibitor PF-04620110 harbor scarce LDs, whereas the DGAT2 inhibitor PF-006424439 only partially blocked LD biogenesis without affecting the cell viability. Combined inhibition of DGAT1 and DGAT2 prevents BVDV-induced LD formation to the same extent as DGAT1 inhibition alone (Figure 2I). Moreover, BVDV infection upregulated *DGAT1* and *DGAT2* mRNA levels (Figure 2J). Finally, we investigated the effect of DGATi on BVDV replication. Compared with the DMSO vehicle, both DGATi inhibitors significantly reduced BVDV replication in a dose-dependent manner at 24 hpi, especially for DGAT1 (Figure 2K). Collectively, these data demonstrate that the block of DGAT enzymatic activity disrupts the biogenesis of LDs, thereby disrupting the BVDV replication.

### BVDV mobilizes LDs by promoting lipolysis rather than lipophagy

**Figure 3:**
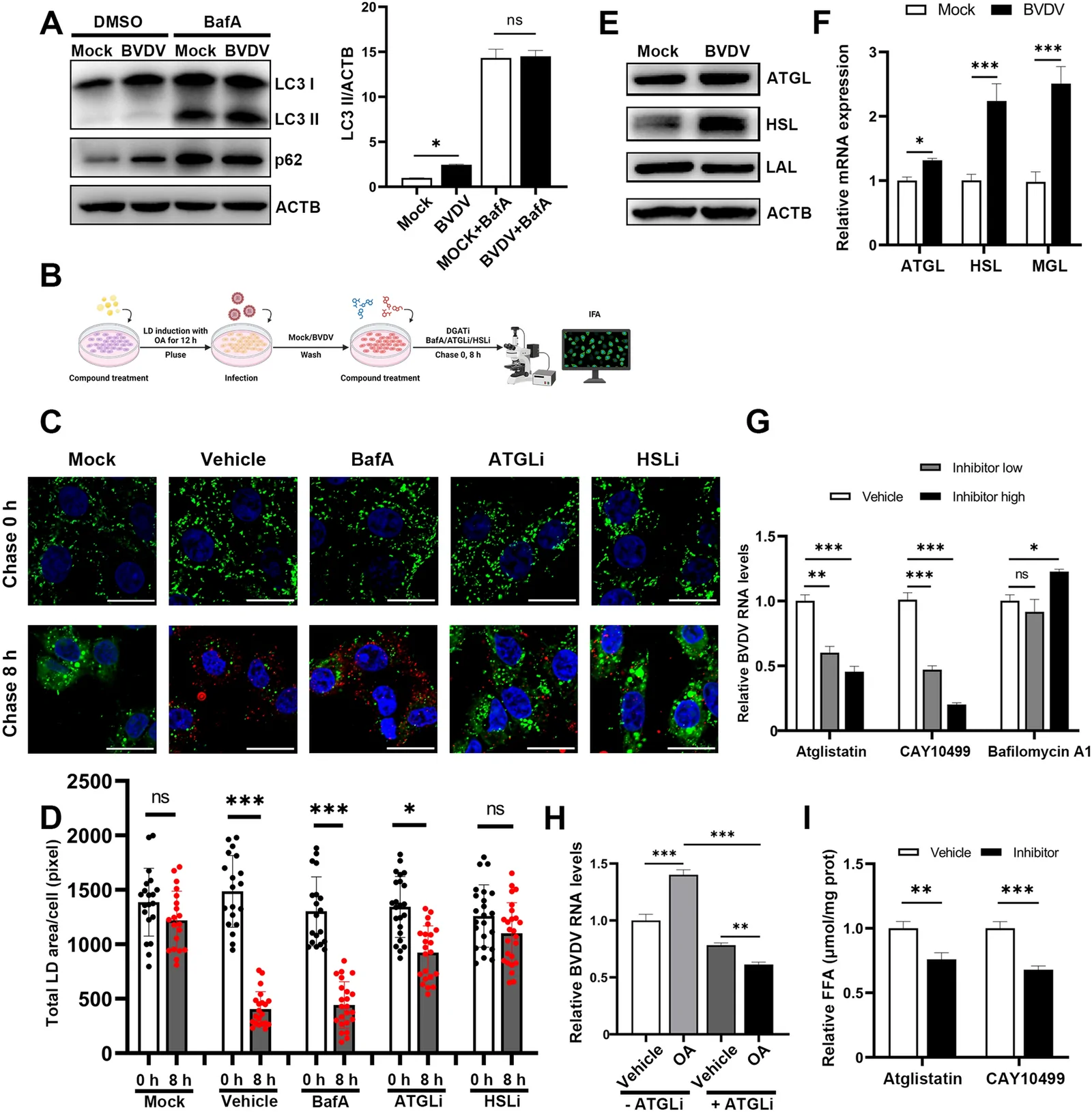
**BVDV mobilizes LDs by promoting lipolysis rather than lipophagy.** In MDBK cells, **A** mock or BVDV-infected (MOI = 5) cells were incubated in DMEM. LC3II and p62 expression were detected by western blot. **B** Schematic of the OA pulse-chase experiment. Cells were pretreated with 200 μM OA for 12 h and were treated with DGAT1 and DGAT2 inhibitors to suppress new LD biogenesis. After 1 h of absorption, mock or BVDV-infected (MOI = 5) cells were incubated at 1 hpi with DMSO or 200 nM bafilomycin A1 or 10 μM Atglistatin or 10 μM CAY10499. **C** Cells were treated as in panel B. At 1 and 9 hpi, cells were fixed and stained with BODIPY 493/503 for LDs (green) and DAPI for cell nuclei (blue). BVDV was detected using the anti-dsRNA (J2) antibody (red). Images are representative of three independent experiments. **D** Cells were treated as in panel B. The total LD area per cell was quantified using the Analyze Particles function of ImageJ (> 20 cells; *n* = 3). (**E–F**) Mock or BVDV-infected (MOI = 5) cells were incubated in DMEM. The expression of key genes of lipolysis and lipophagy was detected by western blot and qRT-PCR. **G** BVDV-infected (MOI = 1) cells were treated with Atglistatin, CAY10499, and bafilomycin A1 for 24 h. Intracellular BVDV RNA at 24 hpi was detected. **H** Cells were pretreated with 200 μM OA for 12 h before BVDV infection (MOI = 1), and cells were incubated at 1 hpi with DMSO or 5 μM CAY10499, respectively. Intracellular BVDV RNA at 24 hpi was detected. **I** Cells were treated as in **E**. Cellular lipids were extracted, and the amount of free fatty acid was quantified. Data are means ± SEM. Statistical significance was determined using unpaired Student’s *t* test or one-way ANOVA (ns, *p* > 0.05; *, *p* < 0.05; **, *p* < 0.01; ***, *p* < 0.001).

Free fatty acids can be released from LDs for utilization by downstream metabolic pathways, including lipolysis and lipophagy [6]. As autophagy plays a proviral role in a diverse range of *flaviviral* infections [26, 27], cells were treated with bafilomycin A1 (BafA), an inhibitor of V-ATPase and lysosomal acidification, to investigate the effects of BVDV on autophagy via immunoblots. Compared with mock infection, BVDV infection leads to the simultaneous accumulation of the autophagosome marker LC3II and the autophagy substrate p62 under the DMSO vehicle at 12 hpi (Figure 3A). Autophagic flux was investigated using a the standard BafA inhibition assay as previously described [28]. As expected, treatment with BafA to block autophagic degradation induced further accumulation of LC3II and p62. After treatment with BafA, the increase in western blot density of LC3-II was significantly lower in the BVDV-infected group than in the mock group, indicating a decrease in net autophagic flux (Figure 3A). These results suggest that BVDV is unlikely to mobilize LDs by promoting lipophagy in the early stages of infection.

To further determine which lipolytic pathway is activated during BVDV infection, we performed an OA pulse-chase experiment (Figure 3B). In this paradigm, MDBK cells were pulsed with 200 μM OA for 12 h to induce LDs and were subsequently treated with DGATi to suppress new LD biogenesis. Cells were then infected with BVDV, with or without various lipolytic inhibitors, and the LD number was assessed by confocal microscopy. The immunofluorescence results demonstrated that, in comparison with the mock group, BVDV infection resulted in a sustained reduction in the number of LDs. However, this did not rescue the persistent reduction in LDs during BVDV infection (Figure 3C–D). These observations argue against lipophagy being responsible for enhanced LD turnover at the early stages of BVDV infection. The next step was to investigate whether lipolysis was responsible for the turnover of LDs. To this end, ATGL and hormone-sensitive lipase (HSL) were pharmacologically inhibited using Atglistatin and CAY10499 [29]. In contrast, we found that Atglistatin and CAY10499 significantly reversed the reduction in LDs caused by BVDV infection (Figure 3C–D), suggesting that BVDV selectively relies on lipolysis to release FAs from LDs. In addition, higher protein and mRNA levels of ATGL and HSL were observed in BVDV-infected cells compared with uninfected cells, whereas the protein level of lysosomal acid lipase (LAL) did not change significantly (Figure 3E–F). These results correlated well with a greater dose-dependent reduction in BVDV replication at 24 hpi when cells were treated with non-toxic concentrations of Atglistatin and CAY10499, but not BafA (Figure 3G).

Given that the increased LDs induced by OA have a proviral effect (Figure 1I), we wanted to further study whether inhibiting lipolytic mobilization of preexisting LDs would affect the proviral effects of OA. Surprisingly, the effect of OA in promoting BVDV replication was completely reversed by Atglistatin, which inhibits the further lipolysis of the LDs after treatment with OA (Figure 3H). Cellular FFA levels were also significantly reduced when cells were treated with CAY10499 and Atglistatin (Figure 3I). In summary, our data demonstrate that BVDV induces the upregulation of ATGL and HSL, thereby promoting lipolysis to facilitate viral replication.

### BVDV enhances the interaction between neutral lipase and LDs instead of increasing LD turnover in lysosomes

**Figure 4:**
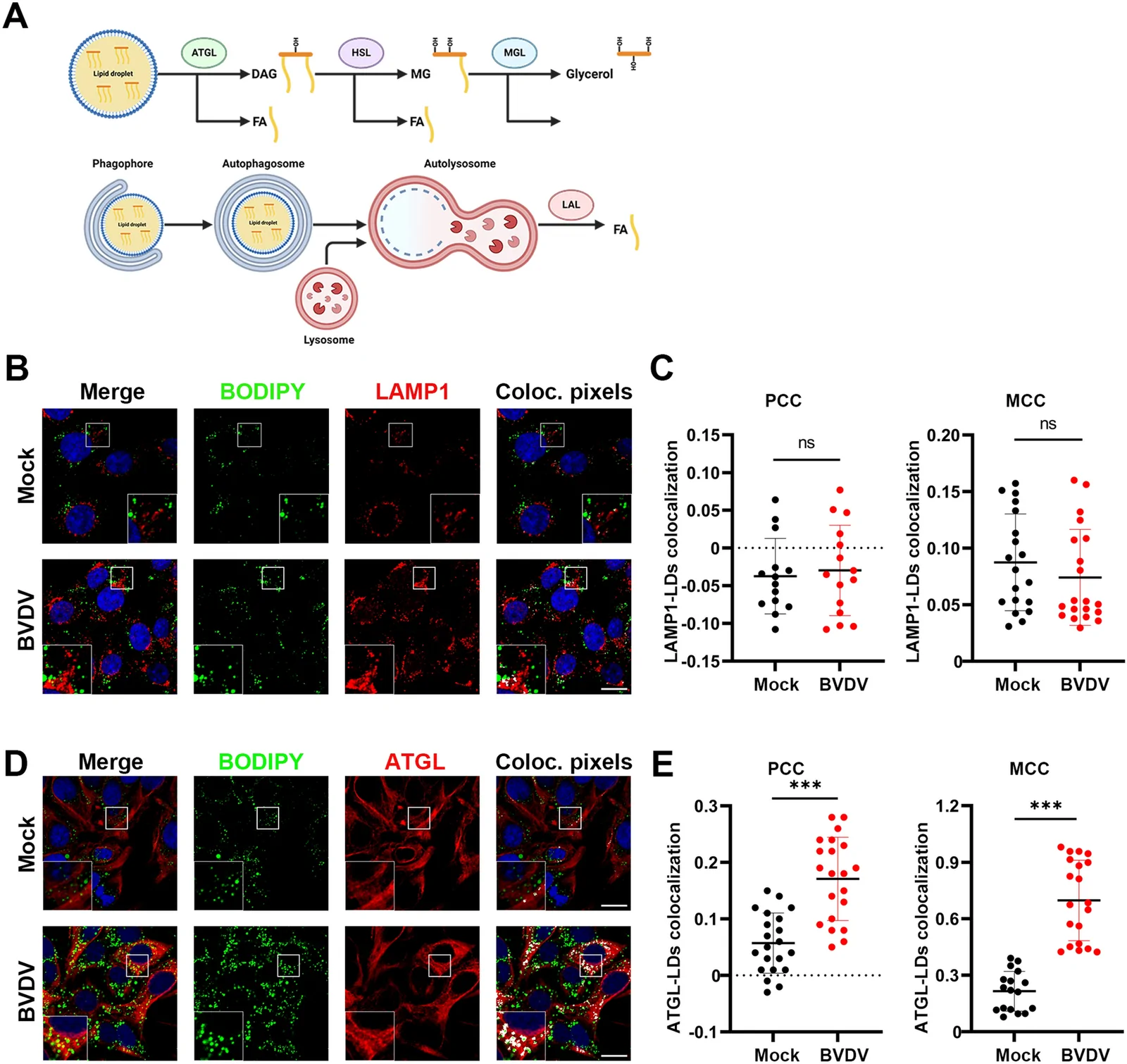
**BVDV enhances the interaction between neutral lipase and LDs instead of increasing LD turnover in lysosomes.** **A** Schematic of lipolysis and lipophagy. **B** Mock or BVDV-infected (MOI = 5) MDBK cells were incubated in a maintenance medium. At 12 hpi, cells were fixed and stained with an anti-LAMP1 antibody to visualize lysosomes (red) and BODIPY 493/503 to visualize LDs (green). The nuclei were stained with DAPI (blue). The rightmost image shows the colocalized pixels as white. **C** Quantification of the colocalization of lysosomes and LDs by calculation of the PCC and MCC between signals in the red and green channels in experimental and control alignments (more than 20 cells; *n* = 3). **D** Mock or BVDV-infected (MOI = 5) MDBK cells were incubated in a maintenance medium. At 12 hpi, cells were fixed and stained with an antibody to ATGL (red) and BODIPY 493/503 to visualize LDs (green). The nuclei were stained with DAPI (blue). The rightmost image shows the colocalized pixels as white. **E** Quantification of the colocalization of ATGL and LDs by calculation of the PCC and MCC between signals in the red and green channels in experimental and control groups (more than 20 cells; *n* = 3).

Lipolysis and lipophagy are mediated by different lipases (Figure 4A). Lipophagy, the delivery of LDs to autolysosomes or lysosomes, is mediated by LAL [30]. Lipolysis, the sequential cleavage of triacylglycerols stored in LDs by lipases recruited to the LDs surface, is mediated by neutral lipases such as ATGL and HSL [31].

We first utilized confocal microscopy to rule out the relationship between LD and lipophagy. In mock-infected cells, LDs and lysosomes scattered in the cytoplasm were well separated, with few LDs in contact with or captured by the lysosomal lumen (Figure 4B). Interestingly, BVDV infection barely alters the association between LDs and lysosomes compared with mock infection (Figure 4B). We quantified the colocalization of LDs and lysosomes using Pearson’s and Mander’s correlation coefficients (PCC, MCC). No significant differences between mock and BVDV-infected groups indicate that even if LDs can be engulfed into autophagosomes, they cannot be further delivered to lysosomes for degradation (Figure 4C).

We then investigated the relationship between LDs and lipolysis-associated neutral lipase by confocal microscopy. ATGL, the first enzyme in the lipolysis pathway, displayed a diffuse cytoplasmic signal and was away from BODIPY-positive structures in mock-infected cells. In BVDV-infected cells, ATGL localized around LDs and showed doughnut-like circular structures (Figure 4D). HSL, the only diacylglycerol lipase involved in lipolysis, follows a similar distribution pattern to ATGL in the context of BVDV infection (Additional file 3A). Quantitative analysis showed that BVDV caused a significant increase in PCC and MCC for both enzymes with colocalization with LDs (Figure 4E, Additional file 3B). Consistent with our OA pulse-chase results, these findings provide compelling evidence that BVDV does not exploit lipophagy, but rather lipolysis, to facilitate LD mobilization in the early stages of infection.

### Redirecting lipid metabolism toward beta-oxidative pathways is critical for BVDV replication

**Figure 5:**
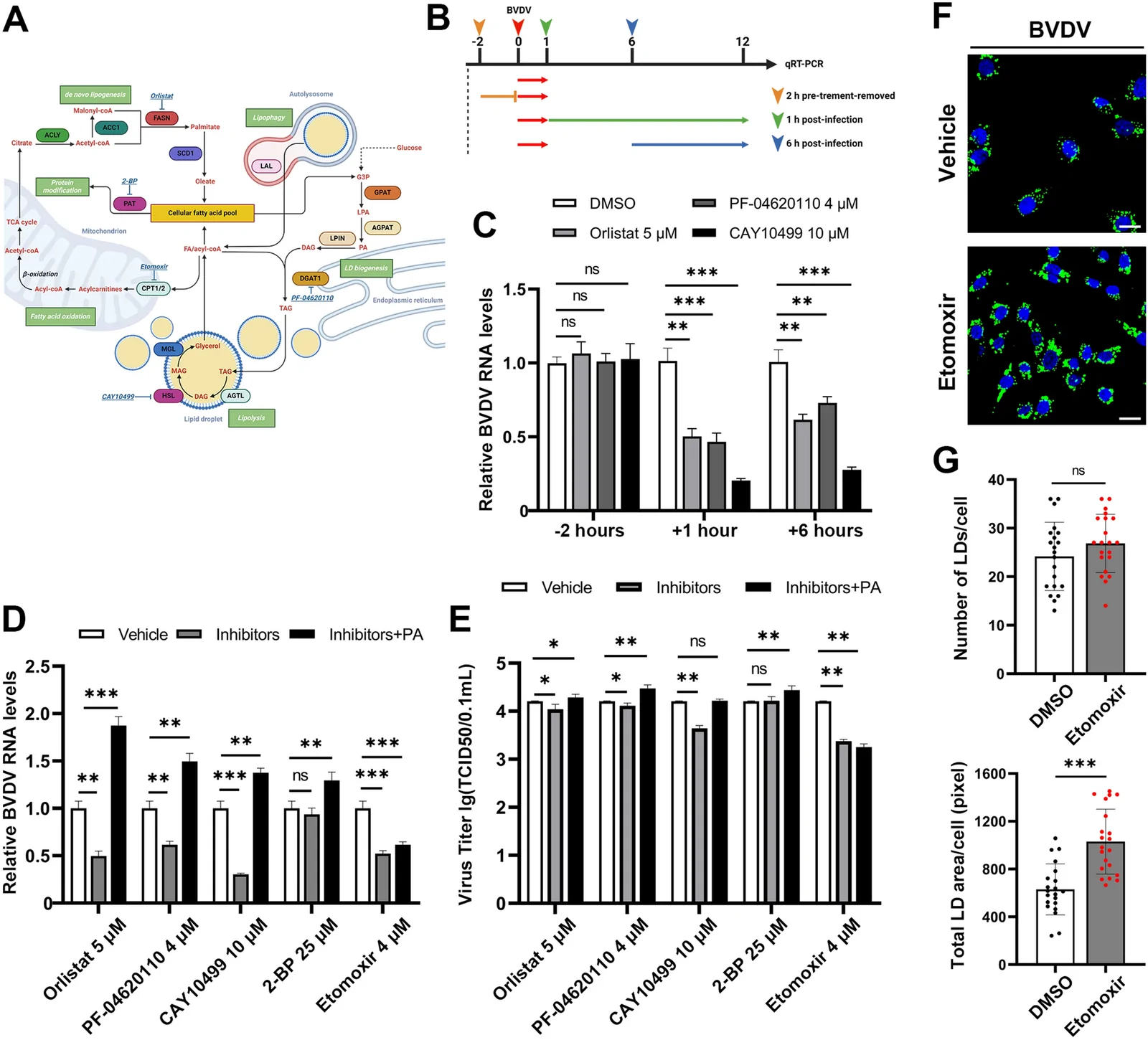
**Regulation of lipid metabolism to beta-oxidative pathways is critical for BVDV replication.** **A** Schematic of neutral lipid metabolism. GPAT, glycerol-3-phosphate acyltransferase; AGPAT, 1-acylglycerol-3-phosphate O-acyltransferase; LPIN, phosphatidate phosphatase LPIN1; G3P, glycerol-3-phosphate; LPA, lysophosphatidic acid; PA, phosphatidic acid; PC, phosphatidylcholine; PS, phosphatidylserine; PE, phosphatidylethanolamine; PI, phosphatidylinositol; 2-BP, 2-bromopalmitate; PAT, palmitoyl acyltransferase. **B** Timeline for the time-of-addition experiment. MDBK cells were either pretreated for 2 h with compounds, which were then removed before infection (−2) or treated at 1 (+1) or 6 (+6) h post infection. **C** qRT-PCR was performed to detect intracellular BVDV RNA levels at 12 hpi. **D** BVDV-infected (MOI = 1) MDBK cells were treated with Orlistat, PF-04620110, CAY10499, 2-BP, Etomoxir, and palmitate as indicated for 24 h. qRT-PCR was performed to detect intracellular BVDV RNA levels at 24 hpi. **E** BVDV-infected MDBK cells (MOI = 1) were treated with inhibitors as described for panel D. TCID_50_ was performed to detect viral titers at 24 hpi. **F** BVDV-infected (MOI = 5) MDBK cells were incubated at 1 hpi with DMSO or 4 μM etomoxir, respectively. At 12 hpi, cells were fixed and stained with BODIPY 493/503 to visualize LDs (green) and DAPI to visualize the cell nuclei (blue). **G** MDBK cells were treated as described for panel F. The average number of LDs per cell and total LD area per cell were quantified using the Analyze Particles function of ImageJ (more than 20 cells; *n* = 3). Data are means ± SEM. Statistical significance was determined using unpaired Student’s *t* test or one-way ANOVA (ns, *p* > 0.05; *, *p* < 0.05; **, *p* < 0.01; ***, *p* < 0.001).

We found that the key steps of lipid metabolism, including de novo lipogenesis, lipid uptake, TAG synthesis, and lipolysis, are critical for viral proliferation (Figure 5A). We then investigated whether the antiviral activity of each compound that inhibits these metabolic steps affected the viral life cycle in a single round of BVDV replication. As indicated in the legend, three conditions were tested: (1) single treatment 2 h before BVDV infection, with compound removed before infection; (2) dosage at 1 hpi until 12 hpi; (3) dosage at 6 hpi until 12 hpi (Figure 5B). Our results showed that Orlistat, PF-04620110, and CAY10499 did not affect the entry stage but significantly inhibited BVDV replication in the post-entry stage (Figure 5C). To further determine whether the antiviral effects of the three inhibitors could be overcome by adding exogenous FAs in multiple rounds of infection, MDBK cells were mock-infected or infected with BVDV at a MOI of 1. After incubating, cells were treated with each pharmacological inhibitor as above for another 24 h to allow multiple cycles of infection, and simultaneously treated with FA-free BSA vehicle alone or BSA-conjugated palmitic acid (PA-BSA) to specifically rescue the phenotype. Compared with the BSA vehicle, which did not affect viral load, PA-BSA completely reversed intracellular BVDV RNA levels at 24 hpi (Figure 5D). Viral supernatants were collected from cells 24 h after treatment with various inhibitors, and then diluted tenfold and used to infect MDBK cells in 96-well plates for viral titer assays. PA-BSA also completely reversed the inhibitory effect of the inhibitor on extracellular viral titers (Figure 5E).

The cellular FA pool has been co-opted by diverse viruses for a variety of purposes, including the palmitoylation of viral proteins and the provision of substrates for energy via FAO [7]. We then preliminarily identified the pathways of FA utilization by BVDV using pharmacological inhibitors. To assess the contribution of palmitoylation to BVDV replication, MDBK cells were infected in the presence of vehicle or the palmitate analogue 2-bromopalmitate (2-BP), an inhibitor of palmitoyl acyltransferases [32], and the infectivity and replication of BVDV were determined. Compared with the DMSO vehicle, 2-BP did not affect BVDV replication at 24 hpi and did not affect cell viability (Figure 5D–E), indicating that palmitoylation does not affect the early stages of the BVDV life cycle. Given that ATGL selectively channels the liberated FAs to oxidative pathways [33], we next tested the role of FAO in BVDV replication by using etomoxir, a compound that targets carnitine palmitoyltransferase 1A (CPT1A), preventing the transport of FFAs into the mitochondria [34]. Interestingly, immunofluorescence analysis showed that etomoxir caused further LD accumulation in BVDV-infected cells without affecting the cell viability (Figure 5F–G). Treatment with etomoxir significantly reduced intracellular BVDV RNA in a way that could not be complemented by replenishing PA-BSA (Figure 5D–E), highlighting the critical role of FAO in the viral life cycle. In summary, our data demonstrate that BVDV induces both lipogenesis and lipolysis, and cellular FAs are transferred into mitochondria to be utilized to facilitate BVDV replication.

### BVDV promotes the proximity of LDs to the mitochondrial network and enhances fatty acid oxidation

**Figure 6:**
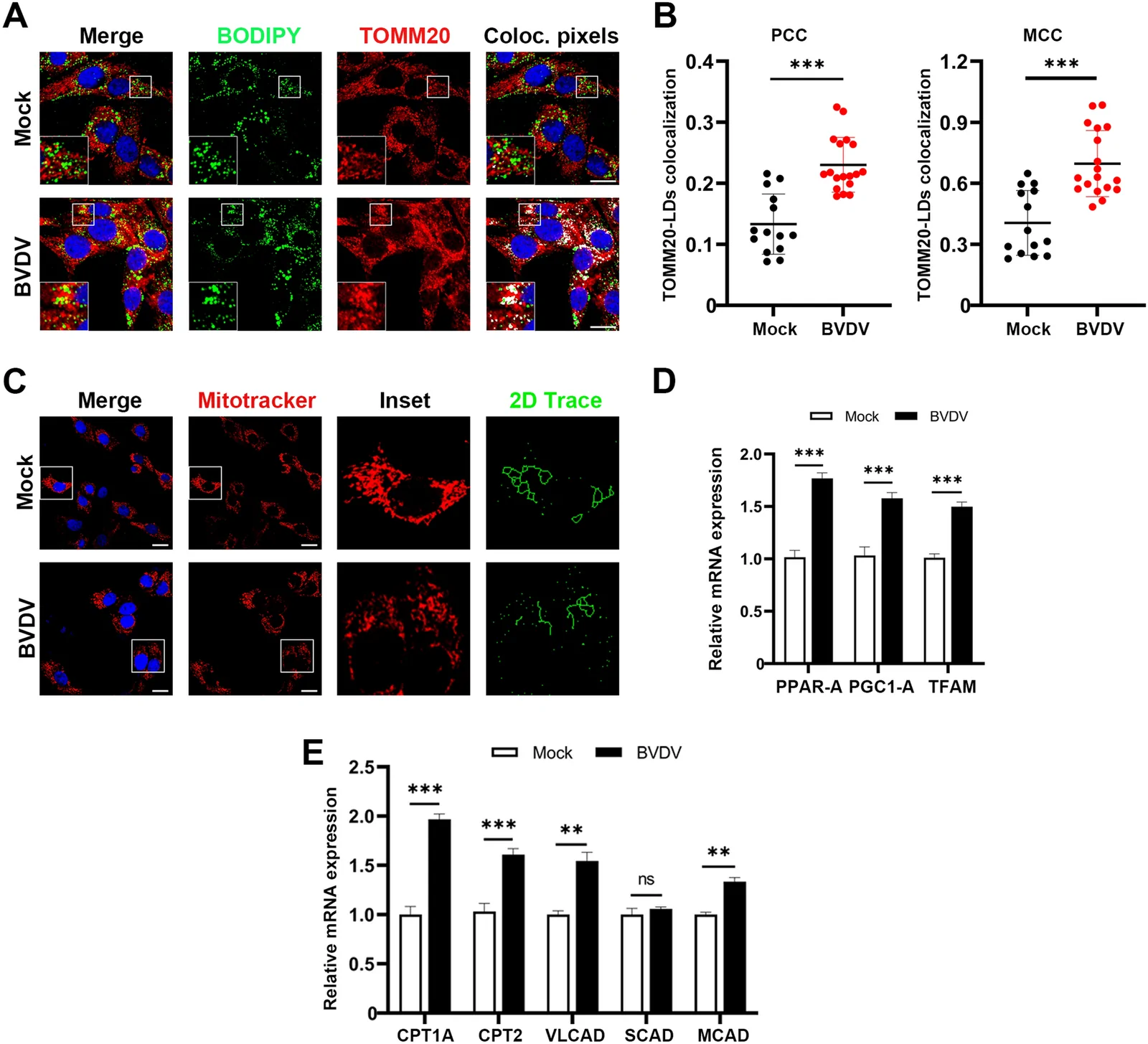
**BVDV promotes the proximity of LDs to the mitochondrial network and enhances fatty acid oxidation.** **A** Mock or BVDV-infected (MOI = 5) MDBK cells were incubated in a maintenance medium. At 12 hpi, cells were fixed and stained with an antibody to TOMM20 (red) to visualize mitochondria and BODIPY 493/503 to visualize LDs (green). The nuclei were stained with DAPI (blue). The rightmost image shows the colocalized pixels as white. Images are representative of three independent experiments. **B** Quantification of the colocalization of TOMM20 and LDs by calculation of the PCC and MCC between signals in the red and green channels in experimental and control groups (more than 20 cells; *n* = 3). **C** Mock or BVDV-infected (MOI = 5) MDBK cells were incubated in a maintenance medium. At 12 hpi, cells were fixed and stained with Mito-Tracker to visualize mitochondria (red) and DAPI to visualize the cell nuclei (blue). The rightmost image represents the 2D trace of the tubular mitochondrial network obtained by the Skeletonize 2D/3D plugin of ImageJ. Images are representative of three independent experiments. **D** MDBK cells were treated as in panel C. qRT-PCR was used to evaluate the expression of key genes involved in mitochondrial biogenesis at 12 hpi. **E** MDBK cells were treated as in panel C. qRT-PCR was used to evaluate the expression of key genes involved in mitochondrial fatty acid beta-oxidation at 12 hpi. Data are means ± SEM. Statistical significance was determined using unpaired Student’s *t* test or one-way ANOVA (ns, *p* > 0.05; *, *p* < 0.05; **, *p* < 0.01; ***, *p* < 0.001).

As dynamic organelles, metabolic crosstalk between mitochondria and LDs via membrane contact sites (MCSs) is well documented in lipid oxidation [35]. Since hepatitis C virus (HCV) and dengue virus (DENV) target both mitochondria and LDs to create a favorable environment for their proliferation [11, 14], we further investigated the effects of BVDV infection on mitochondria-LD contact and FA transfer to better define the cross-talk between mitochondria and LDs. MDBK cells were treated with OA to expand LDs, and the association of mitochondria with LDs was examined using confocal microscopy. Mitochondria were labeled with TOMM20 and LDs with BODIPY 493/503. In mock-infected cells, LDs and mitochondria showed different distribution patterns with limited contact between these two organelles (Figure 6A). In contrast, BVDV-infected cells presented discrete foci where LDs were in close proximity to mitochondria. Despite the colocalization of LDs and mitochondria being only partial, BVDV infection significantly increased the PCC and MCC in comparison with the mock (Figure 6B).

Both viral hijacking and excess lipid accumulation promote mitochondrial dysfunction [36, 37]. Therefore, the impact of BVDV infection on mitochondrial homeostasis was studied. Confocal microscopy showed that mitochondria displayed a round, fragmented morphology as in Figure 6A and detached from the networks in BVDV-infected cells, as obtained by 2D tubular mitochondrial network analysis at 12 hpi (Figure 6C) [38]. In comparison, mock-infected MDBK cells displayed a normal mitochondrial network (Figure 6C). Consistent with the abnormal mitochondrial network, fluorescence microscopy with the mitochondrial membrane potential sensitive dye JC-1 showed a marked increase in green monomers in BVDV-infected cells, indicative of mitochondrial depolarization [39]. In contrast, mock-infected cells displayed predominantly red aggregates, reflecting normal mitochondrial membrane potential (Additional file 4). We next investigated the status of mitochondrial biogenesis during BVDV infection. qRT-PCR analysis showed that the transcript levels of biogenesis-related transcriptional activators and cofactors (PPAR-A, PGC1-A, and TFAM) were upregulated in BVDV-infected cells at 12 hpi due to BVDV (Figure 6D) [40]. Meanwhile, BVDV upregulated the mRNA levels of FAO-related genes [41]. CPT1 and CPT2, which are responsible for the carnitine cycle, as well as Acyl-CoA dehydrogenases (e.g., VLCAD, MCAD, and SCAD) were also upregulated in response to BVDV (Figure 6E). In summary, by co-opting mitochondrial biogenesis, BVDV could sustain proper mitochondrial FAO function in the context of mitochondrial dysfunction, thereby completing its life cycle, and additionally, BVDV promotes LD-mitochondrial interactions and facilitates mitochondrial biogenesis and FAO function.

### BVDV accelerates the transfer of FAs from LDs to mitochondria

**Figure 7:**
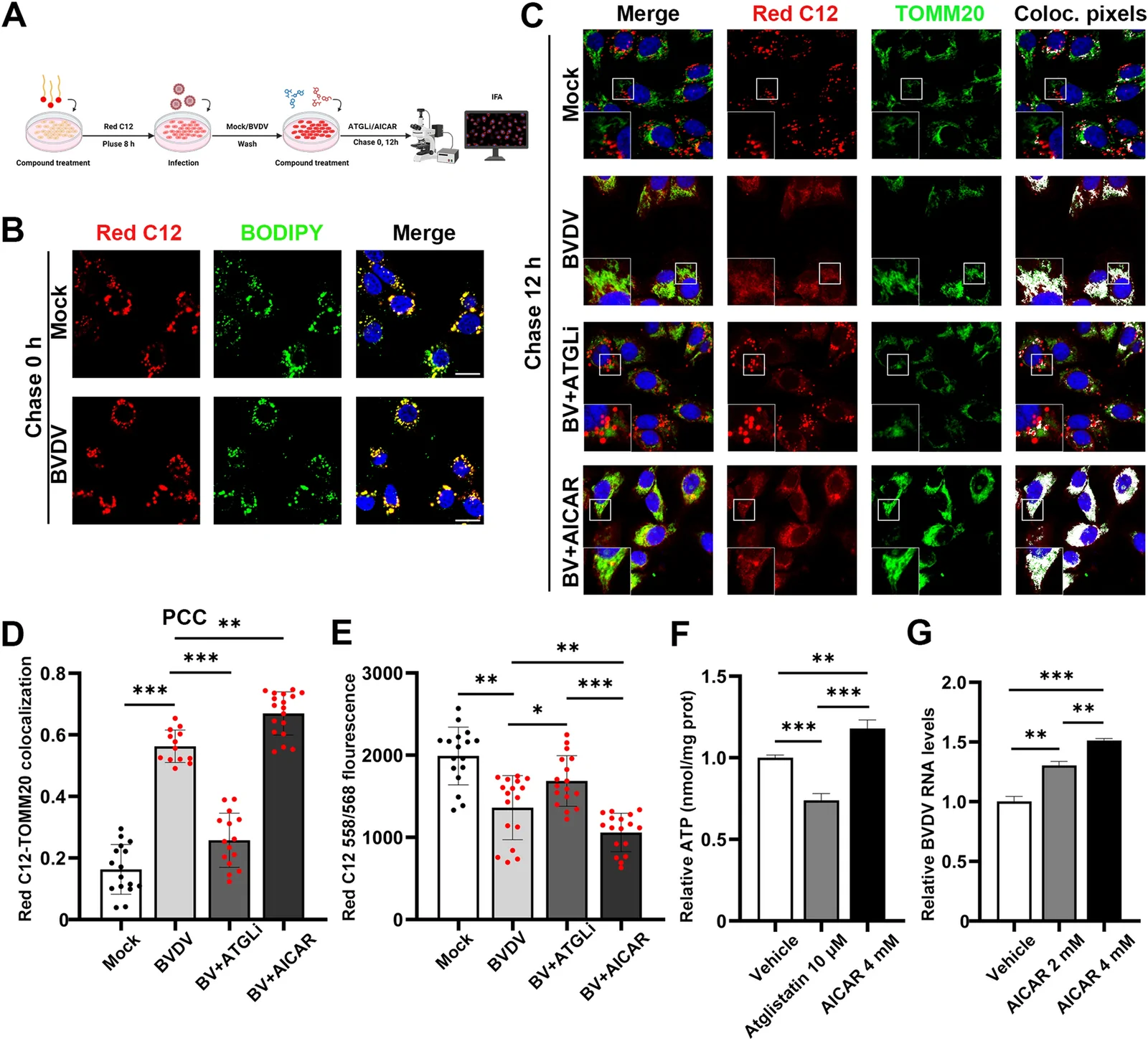
**BVDV accelerates the transfer of FAs from LDs to mitochondria. A** Schematic representation of the Red C12 pulse-chase assay. MDBK cells were pulsed with Red C12 for 8 h. After 1 h of absorption, BVDV-infected (MOI = 5) MDBK cells were incubated at 1 hpi with DMSO, 10 μM Atglistatin, or 4 mM AICAR, respectively. Cells were then chased in DMEM with 1% FBS for 12 h and imaged to determine the subcellular FAs localization. **B**–**C** MDBK cells were treated as described in panel A. At 0 hpi, cells were fixed and stained with BODIPY 493/503 for LDs (green) and DAPI for cell nuclei (blue). **C** At 12 hpi, confocal fluorescence microscopy of MDBK cells stained with TOMM20 for mitochondria (green) and DAPI for cell nuclei (blue). The rightmost image shows the colocalized pixels as white. **D** Quantification of the colocalization of Red C12 and mitochondria by calculation of the PCC between signals in the red and green channels in experimental and control groups (> 20 cells; *n* = 3). **E** Total Red C12 fluorescence per cell was quantified using ImageJ (> 20 cells; *n* = 3). **F** MDBK cells were pulsed with Red C12 for 8 h. After 1 h of absorption, BVDV-infected (MOI = 5) MDBK cells were incubated at 1 hpi with DMSO, 10 μM Atglistatin, or 4 mM AICAR. Cells were then chased in DMEM supplemented with 1% FBS for 12 h, and the ATP amount was quantified. **G** MDBK cells were treated as in **F**. After 1 h of absorption, BVDV-infected (MOI = 1) cells were treated with AICAR for 24 h. Intracellular BVDV RNA at 24 hpi was detected. Images are representative of three independent experiments. Data are means ± SEM. Statistical significance was determined using unpaired Student’s *t* test or one-way ANOVA (ns, *p* > 0.05; *, *p* < 0.05; **, *p* < 0.01; ***, *p* < 0.001).

Given that BVDV infection increases LD–mitochondrial interactions and upregulates the expression of FAO-related genes, we used a pulse-chase assay with the saturated fluorescent FA analogue BODIPY 558/568 C12 (Red C12) to investigate whether BVDV plays a role in the translocation of FAs (Figure 7A). At the beginning of the chase period (*t* = 0 h), bright Red C12 dots in both BVDV- and mock-infected cells colocalized with BODIPY 493/503 (Figure 7B).

In mock-infected cells, almost all Red C12 signals were retained in LDs throughout the chase period (*t* = 12 h) (Figure 7C). In contrast, BVDV-infected cells showed a dramatic loss of Red C12 signal from LDs, with a marked redistribution of signal into mitochondria, resulting in almost complete overlap of Red C12 with TOMM20. The ATGL-specific inhibitor Atglistatin significantly blocked the transport of Red C12 from LDs to mitochondria induced by BVDV, indicating that lipolysis plays an important role in directing fatty acids towards oxidation.

AICAR, a pharmacological activator of fatty acid beta-oxidation [42], was able to further promote FA transfer from LDs to the mitochondria in BVDV-infected cells without affecting cell viability (Figure 7C). Quantitative results of PCC confirmed these findings (Figure 7D).

Moreover, BVDV caused a progressive decrease in total fluorescence intensity of intracellular Red C12 at 12 hpi, which was reverted by Atglistatin and further boosted by AICAR (Figure 7E). FAs are transferred from LDs to mitochondria for ATP production. We found that Atglistatin decreased ATP levels compared with those in BVDV-infected cells, whereas AICAR increased ATP levels (Figure 7F). Finally, AICAR significantly promoted BVDV replication in a dose-dependent manner at 24 hpi (Figure 7G). In conclusion, these findings indicate that BVDV promotes mitochondrial-LD interactions and facilitates lipolysis-dependent LD-to-mitochondria FAs transport for ATP production.

### BVDV core protein targets LDs to promote the mobilization of LDs

**Figure 8:**
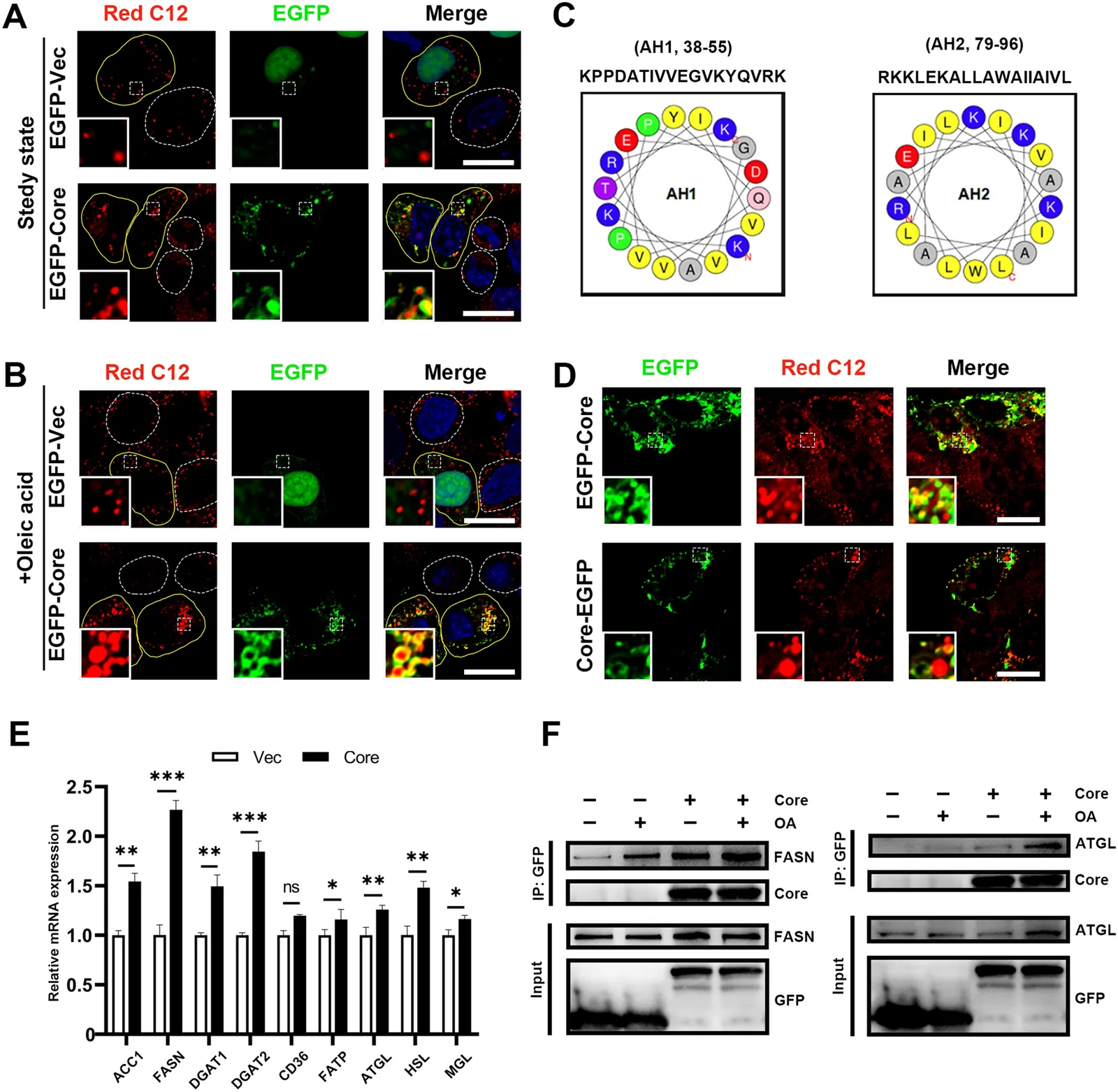
**BVDV core protein targets LDs to promote the mobilization of LDs. A** Cells were transfected with pEGFP-C1 or pEGFP-core plasmid for 12 h, and LDs were labeled with Red C12 (red). At 24 hpi, cells were fixed and stained with DAPI to visualize the nuclei (blue). Images are representative of three independent experiments. **B** Cells were treated as in panel A and treated with 200 μM OA for another 12 h. LDs were labeled with Red C12 (red). At 24 hpi, cells were fixed and stained with DAPI to visualize the cell nuclei (blue). Images are representative of three independent experiments. **C** The two predicted amphipathic helical domains of residues 38–55 (AH1) and residues 79–96 (AH2) of BVDV core protein were generated via the HeliQuest tool. **D** Cells were transfected with pcore-EGFP or pEGFP-core plasmid for 12 h and treated with 200 μM OA for another 12 h. LDs were labeled with Red C12 (red). At 24 hpi, cells were fixed and stained with DAPI to visualize the cell nuclei (blue). Images are representative of three independent experiments. **E** Cells were treated as in panel A. qRT-PCR was used to evaluate the expression of key genes involved in lipid metabolism at 24 hpi. **F** Cells were transfected with pEGFP-C1 or pEGFP-core plasmid for 12 h and treated with 200 μM OA for another 12 h. At 36 h, protein interactions were detected by immunoprecipitation using anti-EGFP magnetic agarose beads and immunoblotting analysis with FASN and ATGL antibodies. Data are means ± SEM. Statistical significance was determined using unpaired Student’s *t* test or one-way ANOVA (ns, *p* > 0.05; *, *p* < 0.05; **, *p* < 0.01; ***, *p* < 0.001).

Given that the core proteins of both HCV and DENV can target LDs [10, 11], we investigated whether the BVDV core protein has a similar function. We first examined the colocalization of Red C12-labeled LDs with EGFP-tagged core under steady state or during LD biogenesis induced by OA treatment for 12 h using confocal microscopy. The empty vector fluorescence was widely distributed throughout the cell and did not colocalize with LDs in either state. Whereas in a steady state, EGFP-core was punctate and partially colocalized with LDs (Figure 8A), upon OA treatment, EGFP-core was remarkably targeted to LDs (Figure 8B). To better understand the LD targeting mechanism of the core protein, we used the HeliQuest tool to identify amphipathic helices of the BVDV core protein [43]. The BVDV core protein is a 102-amino-acid-long peptide containing the first amphipathic helix (AH1), ranging from residues 38 to 55, and the second amphipathic helix (AH2), ranging from residues 79 to 96 (Figure 8C). The secondary structure predictor PSIPRED and RoseTTAFold protein structure prediction also support the hypothesis of the presence of an amphipathic helix (AH2) at the C-terminus of the core protein (Additional file 5A–B). We next expressed EGFP-tagged core at the C-terminus (core-eGFP) and found that core-EGFP did not localize to lipid droplets (LDs) as distinctly as EGFP-core, suggesting that AH2 may play a crucial role in the localization of the core protein to LDs (Figure 8D). In addition, the colocalization pattern of EGFP-tagged core with LDs, which we labeled with Nile Red [44], was consistent with the above results (Additional file 5C–D).

We observed that core protein transient expression led to a significant induction of LDs relative to empty vector in both steady state and OA-treated cells, thus we hypothesized that the core protein may play a direct role in the biogenesis and degradation of LDs. We next used qRT-PCR to determine the mechanism by which core influences LD abundance. Consistent with observations in BVDV-infected cells, core overexpression significantly upregulated the RNA levels of lipogenic genes (*ACLY*, *ACC1*, and *FASN*) and esterification-related genes (*DGAT1*, *DGAT2*), while core expression did not affect the RNA levels of FA uptake genes (*CD36*, *FATP*) (Figure 8E), suggesting that BVDV increases de novo lipogenesis through core proteins and that other viral proteins regulate FA uptake. Overexpression of core also increased levels of lipolysis-related genes (*ATGL*, *HSL*, and *MGL*) and inhibited autophagy by decreasing LC3II and increasing p62 protein levels (Figure 8E, Additional file 5E), suggesting that BVDV promotes lipolysis rather than lipophagy through its core protein. Finally, we investigated the possibility that the core protein regulates de novo lipogenesis and lipolysis by interacting with key enzymes. To this end, EGFP-core was expressed in bovine cells, and protein interactions were examined by immunoprecipitation. We found that core protein specifically interacted with endogenous FASN and ATGL compared with the empty vector, while OA treatment further increased their interaction with core protein (Figure 8F). Furthermore, the core protein increased the interaction with the mitochondrial outer membrane protein TOMM20 compared with the EGFP empty vector (Additional file 5F).

**Figure 9:**
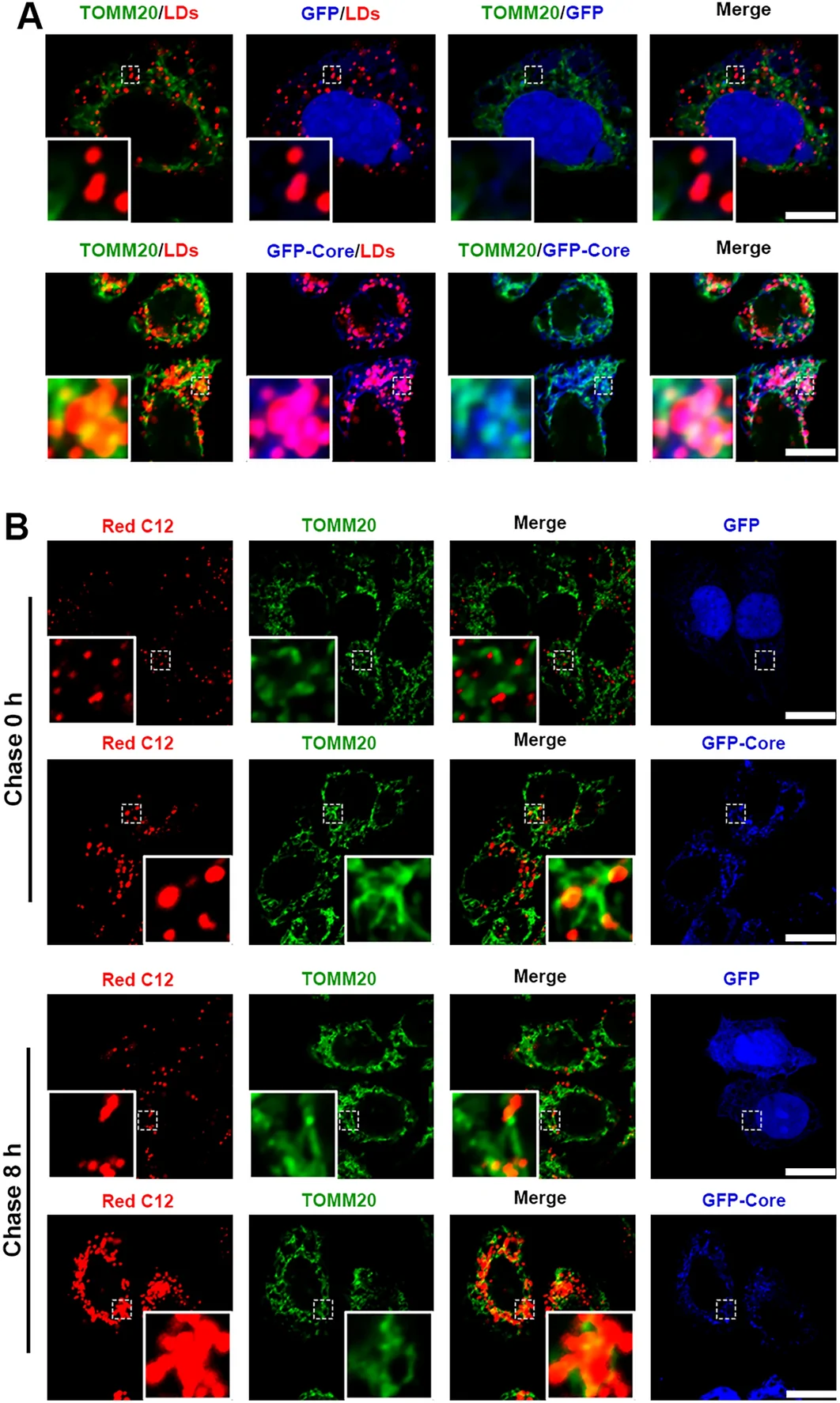
**BVDV core protein links mitochondria to LDs.** **A** Cells were transfected with pEGFP-C1 or pEGFP-core (blue) plasmid for 12 h, and LDs were labeled with Red C12 (red). At 24 hpi, cells were fixed and stained with anti-TOMM20 to visualize the mitochondria (green). Images are representative of three independent experiments. The scale bar represents 20 μm. **B** Pulse-chase assays were performed in vector- or EGFP-core-expressing cells. HeLa cells were incubated in complete medium containing 1 μM BODIPY 558/568 C12 (red) for 16 h. Cells were washed with PBS three times and then chased (0 h) or no-chased in EBSS for 8 h, and then were fixed and stained with anti-TOMM20 (green). Images are representative of three independent experiments. The scale bar represents 20 μm.

Compared with vector-transfected cells, core protein expression not only increased colocalization between core and mitochondria, but also between core and LDs, thereby bringing mitochondria and LDs into proximity (Figure 9A). Moreover, the transfer rate of Red C12 from LDs to mitochondria was significantly higher in EGFP-core-expressing cells compared with vector-transfected cells (Figure 9B).

**Figure 10:**
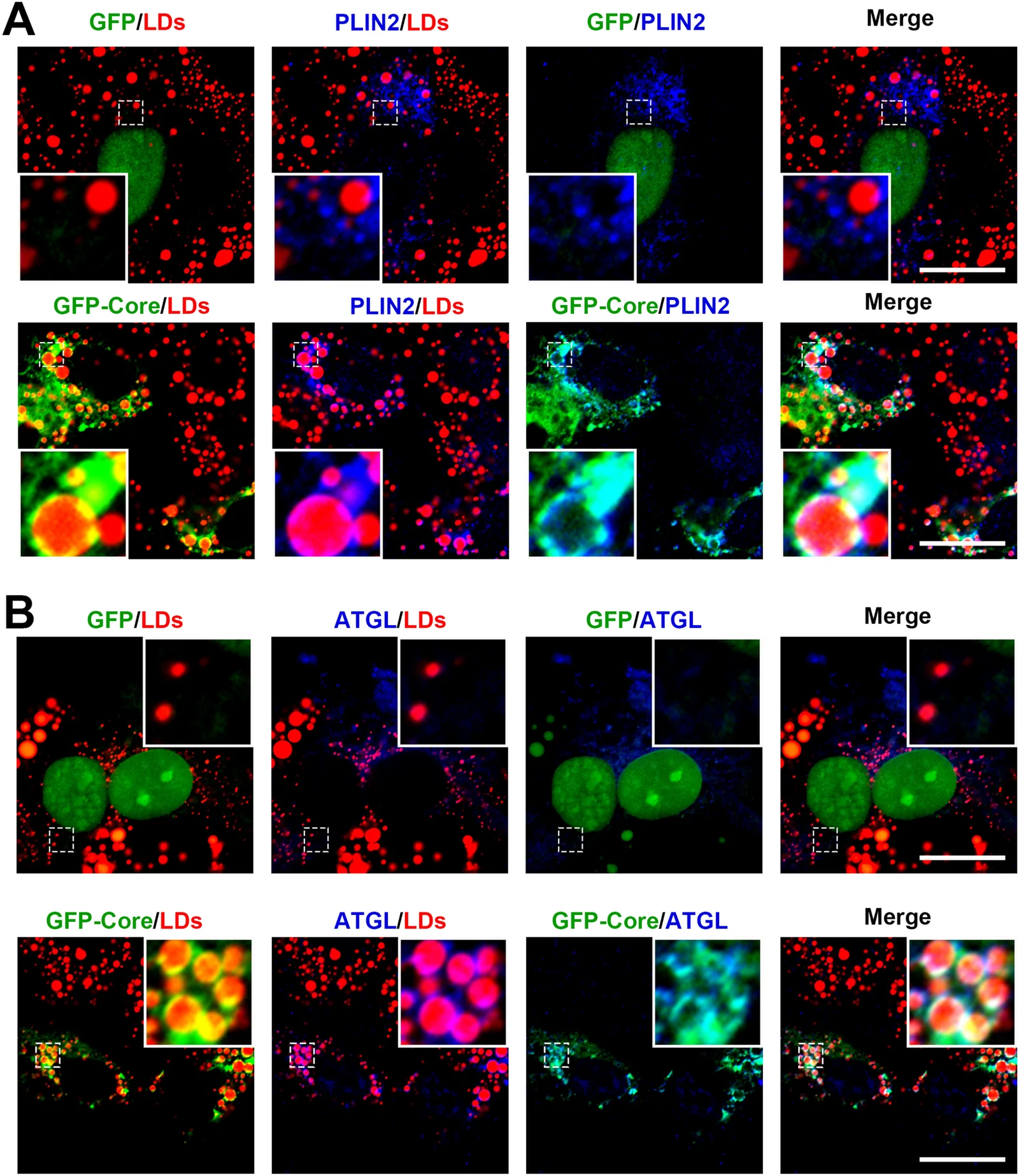
**Core recruits ATGL to the surface of lipid droplets.** **A** Co-transfection of cells with p-EGFP-C1/pEGFP-core and pCMV-PLIN2-FLAG for 12 h, and LDs labeled with Red C12 (red). At 24 hpi, cells were fixed and stained with a fluorescent secondary antibody to detect PLIN2 (blue). Images are representative of three independent experiments. **B** Co-transfection of cells with p-EGFP-C1/pEGFP-core and pCMV-ATGL-HA for 12 h, and LDs labeled with Red C12 (red). At 24 hpi, cells were fixed and stained with a fluorescent secondary antibody to detect ATGL (blue). Images are representative of three independent experiments.

To further clarify the functional significance of core-mediated interactions with lipid metabolic enzymes, we investigated whether core facilitates lipolysis by recruiting ATGL to LDs, given that free fatty acids released by ATGL-mediated lipolysis are preferentially directed toward fatty acid oxidation (FAO). To this end, we co-transfected cells with p-EGFP-core and pCMV-PLIN2-FLAG to confirm LD surface localization, and with p-EGFP-core and pCMV-ATGL-HA to assess ATGL recruitment. The results showed that the surface of Red C12 lipid droplets transfected with empty vectors was enveloped by PLIN2, whereas core was significantly localized to the surface of Red C12 lipid droplets compared with empty vectors, forming a colocalized ring-like structure with PLIN2 on the lipid droplet surface, suggesting that core is indeed localized to the surface of lipid droplets and follows the same distribution pattern as PLIN2 (Figure 10A). In cells transfected with the empty vector, the neutral lipase ATGL was scattered throughout the cytoplasm and showed almost no colocalization with Red C12 lipid droplets (Figure 10B). In contrast, while core is directed to the surface of lipid droplets, it also recruits ATGL to the surface of lipid droplets, where core and ATGL together form a crescent-shaped structure (shown as white pixels in the figure) (Figure 10B), confirming that core can recruit ATGL to the surface of lipid droplets to release fatty acids, thereby accelerating the LD-mitochondrial transfer of lipids.

In summary, our data suggest that the BVDV core protein targets the surface of lipid droplets, where it recruits key enzymes involved in lipid metabolism: fatty acid synthase (FASN) and adipose triglyceride lipase (ATGL). The core protein also interacts with mitochondria, thereby linking them to LDs and increasing the efficiency of FAO.

## Discussion

Lipid droplets (LDs) are closely associated with *flaviviral* infection, but little is known about how BVDV utilizes LDs. In this study, we revealed novel mechanisms by which BVDV targets LDs to reprogram cellular metabolism for viral replication: (i) BVDV promotes the accumulation of cellular LDs by enhancing de novo lipogenesis, accelerating exogenous fatty acid uptake, and increasing esterification of FAs; (ii) BVDV promotes lipolysis to release FFAs and increase the cellular FAs pool to promote replication; (iii) BVDV promotes LD–mitochondrial interaction and accelerates FAs transfer to mitochondria for FAO; and (iv) BVDV core protein targets the surface of LDs, increasing and recruiting FASN and ATGL to promote LD mobilization.

LDs are highly dynamic storage organelles at the center of lipid and energy homeostasis [45]. In addition to a major role in energy storage, LDs are also important cellular hubs that traffic proteins and lipids between organelles, regulate ER stress, and contribute to viral infections [5, 7]. Previous studies have shown that several RNA viruses enhance lipid droplet formation in the early stages of infection and block lipid biosynthesis inhibiting viral replication [46, 47]. At the same time, other viruses utilize lipolysis or lipophagy to break down LDs in the mid-stage of infection to promote viral replication [48, 49]. Our study showed that BVDV caused an early LD formation and late breakdown during its life cycle. This dynamic change manipulated by BVDV may serve as a common feature of RNA viruses, indicating that LDs play different roles at different stages of infection. It is therefore necessary to determine whether the biogenesis of LDs is beneficial for BVDV replication. Our results suggest that the accumulation of LDs induced by treatment with OA plays a role in promoting viral replication during BVDV infection. The size of LDs also varies with lipid accumulation, and these heterogeneities in size may result in different LD populations performing distinct functions [50]. In contrast to the significant increase in number and area, the mean diameter of LDs remained at approximately 1.4 μm after infection. LD size depends on the net result of nascent LD biogenesis and mature LD degradation. To better investigate the effect of BVDV infection on LD dynamics, subsequent experiments could employ GPAT4, a membrane marker for nascent LDs [51], and HSD17B11, a marker for mature LDs [52], to validate changes in different LD populations.

The increased LD number induced by BVDV could be due to increased intracellular FA production and esterification, decreased lipid hydrolysis from LDs, or a combination of the above two situations. As a barrier to maintaining LD homeostasis, PLIN2 (also known as ADRP) and PLIN3 (also known as TIP47) were recruited to the surface of LDs during their biogenesis [16]. With LD maturation, PLIN3 is progressively displaced and remains stable in the cytoplasm [53]. The mRNA levels of these PLINs in this study increased significantly with infection, especially that of *PLIN3*, suggesting that BVDV likely promotes overall LD biogenesis.

Triacylglycerols required for LD biogenesis are primarily derived from exogenously circulating lipids and endogenously synthesized FAs. Through DNL, FAs are synthesized from acetyl-CoA generated from glucose, glutamine, or acetate. This acetyl-CoA is then synthesized into palmitate and further elongated and desaturated through a series of enzymatic reactions [54]. Together with FAs taken up from the environment, they form a complex pool of substrates for the synthesis of FA-containing lipids. DNL induced by viral infection can reduce viral dependence on externally supplied lipids [55]. We first assessed the DNL capacity of BVDV laterally by excluding exogenous lipid availability in FA-free medium. The mRNA levels of the major lipogenic genes were all increased significantly by BVDV infection, suggesting that BVDV fully activates the DNL pathway at the transcriptional level. Studies have shown that viruses depend on the selected pathways, such as estimated glomerular filtration rate (eGFR), transforming growth factor-beta (TGF-β), and SREBP-1 pathways, to promote LD production [56, 57]. Our data suggest that BVDV infection activates certain relevant transcription factors, and subsequent experiments could employ the luciferase assay to further investigate the transcriptional mechanisms responsible for BVDV induced lipogenesis.

It is important to note that the FASN inhibitor Orlistat may cause side effects such as the accumulation of malonyl-CoA or acting on other unidentified targets. Accumulation of malonyl-CoA can further lead to protein malonylation or cytotoxicity [58]. We ruled out these nonspecific effects by back-complementation assays, but the ability of PA-BSA to completely reverse the antiviral effects of Orlistat suggests that BVDV can use exogenous lipids. Metabolic flexibility allows cells to compensate for disrupted lipid homeostasis by switching to the uptake of exogenous FAs in states that limit lipid synthesis, such as hypoxia or nutrient deprivation [59]. We confirmed the increase in exogenous lipid uptake by BODIPY 500/510. However, changes in the expression of several enzymes responsible for FA uptake were not consistent. These observations suggest that antiviral therapy using FASN inhibitors needs to be considered in conjunction with reducing the availability of exogenous FA and investigating the molecular mechanisms responsible for lipid uptake.

Diacylglycerol acyltransferase enzymes (DGATs) mediate the final committed step in TAG synthesis. DGAT1 is completely ER-resident, whereas DGAT2 localizes to both the ER and around LDs. These differences in location may represent distinct functions in LD biogenesis. DGAT1 is mainly responsible for incorporating exogenous FAs into TAG or re-esterifying FAs after lipolysis, whereas DGAT2 is thought to play a major role in esterifying de novo synthesized FAs [60]. Considering the efficiency of DGAT1 inhibition alone and the importance of DGAT1 for preventing mitochondrial dysregulation and ER stress under lipolytic conditions [37], we used only PF-04620110 to inhibit new LD biogenesis in subsequent pulse-chase experiments to explore the possibility of LD degradation.

Considering the kinetic changes in LDs during BVDV infection with a sustained increase followed by a decrease at 24 hpi, we hypothesize that LD degradation occurs concurrently with LD synthesis during the early stages of BVDV infection, but is masked by the intense LD biogenesis. Existing studies on *flavivirus* initially led us to explore the role of lipophagy in the mobilization of LDs [26, 27]. However, either an increase in autophagosome formation or a decrease in autophagosome degradation increased LC3II levels, but in our case this increase was accompanied by an accumulation of p62, suggesting that autophagic degradation was blocked in the context of BVDV infection. Consistent with our LC3 II turnover results, OA pulse-chase experiments and unchanged LAL protein levels together exclude the contribution of lipophagy to the mobilization of LDs by BVDV. More recent studies have demonstrated that lipolysis acts as an upstream regulator of lipophagy in hepatocytes, facilitating the breakdown of larger LDs into smaller LDs [61]. Our experiments confirmed that lipolysis degraded LDs in a state of lipophagy inhibition, but the HSL-specific inhibitor CAY10499 was more efficient in limiting LD production and viral replication compared with Atglistatin. ATGL is a well-characterized major TAG lipase in adipose tissue and heart, but additional TAG lipases in non-adipocyte tissue function redundantly to ATGL and compensate for its inhibition [62]. HSL is the only diacylglycerol lipase that cleaves diacylglycerol to monoacylglycerol and free FAs. Although confocal results showed that BVDV increased the interaction between LD and these two neutral lipases at the same time, inhibition of the enzymatic activity of HSL had a more potential effect on prohibiting BVDV replication. CAY10499 causes aggregation of LDs with concomitant impairment of viral replication, but this is distinct from the LD-dependent enhancement of BVDV replication by OA. Meanwhile, CAY10499 was able to completely reverse the effect of OA in promoting BVDV replication. These data suggest that increasing the number of LDs does not always support BVDV replication, as FAs released from lipolysis are critical for viral replication.

Studies have shown that severe acute respiratory syndrome coronavirus 2 (SARS-CoV-2) and DENV promote replication via cellular beta-oxidation [49, 63], whereas HCV-induced lipid oxidation is detrimental to the viral life cycle and might be part of a host metabolic antiviral response [34]. We then used etomoxir to confirm the effect of fatty acid oxidation on viral replication. Etomoxir also causes aggregation of LDs with concomitant impairment of viral replication, and this inhibition could not be fully rescued by PA-BSA. It is reasonable to speculate that the liberated FAs must be transferred to the mitochondria to exert their proviral effect.

Although the biochemical basis of FA import into mitochondria has been extensively studied, how FAs are transferred from LDs to mitochondria to drive FA oxidation is still not fully understood. According to current models, FAs transfer most likely occurs at mitochondria-LD MCSs, where the membranes of the two opposing organelles are in close proximity (typically 10–30 nm) [64]. This spatial proximity would reduce the accumulation of cytosolic-free FAs, preventing lipotoxicity and/or aberrant lipid signaling. Mitochondrial-LD interactions induced by BVDV infection are thus hypothesized to provide a bridge for the transfer of FAs to mitochondria, but direct transfer remains difficult to demonstrate. To enable visualization of FA transfer from LDs to mitochondria, we utilized a widely characterized fluorescent FA analog-based pulse-chase assay by using BODIPY-C12 (Red C12) and adapting it to specific details [9]. It should be noted that Red C12 has different biophysical properties than endogenous FAs, thus affecting their transport and metabolism and producing nonspecific results [65]. Therefore, the FA transport result should be further verified in the subsequent experiments by using isotope-labeled FAs.

Mitochondrial homeostasis is critical for FAO function. In this study, we observed a considerable upregulation in the mRNA levels of enzymes responsible for both the carnitine cycle and dehydrogenation in response to an increase in the cellular fatty acid pools within BVDV-infected cells. Excessive fatty acid oxidation during viral infection may lead to mitochondrial dysfunction [37]. Our results demonstrated that BVDV infection deficiency resulted in abnormal mitochondrial morphology and dissociation of the mitochondrial network. Thus, the BVDV-induced increase in transcription factors associated with mitochondrial biogenesis could compensate for this impairment.

The association between +ssRNA viral proteins and LDs is particularly strong: HCV and DENV capsid proteins colocalize with LDs [10, 11]; Poliovirus protein 2BC is cleaved into 2B and 2C and targeted to LDs [29]; and coronavirus ORF6 protein is targeted to LDs and localizes at the mitochondrial–LD contact site [63]. Viral proteins utilizing amphipathic helices have been identified to target LDs, and depletion of amphipathic helices abolished LD association [66]. In this study, we found that there are two amphipathic helices in the core protein and that the C-terminal helix may be primarily responsible for direct insertion into the LD lipid monolayer. To determine whether the two amphiphilic helices were required for core-LD binding, subsequent experiments required the introduction of point mutations at the hydrophobic interface of each helix to disrupt their structure.

Viral proteins can physically interact with the lipogenesis and lipolysis machinery [63]. In our experiments, the BVDV core protein interacts with FASN, ATGL, and TOMM20. Given that antiviral strategies often focus on directly targeting viral proteins [67], further experiments will require the construction of truncated mutants and the application of in vitro GST pull-down assays to further confirm the direct interactions between core and host proteins, laying the groundwork for the development of broad-spectrum antiviral strategies targeting host factors critical for virus replication.

Although our study demonstrates that BVDV exploits lipid droplet metabolism to facilitate viral replication during the early stage of infection, several limitations remain. First, our study mainly focused on the role of lipid droplet biogenesis and lipolysis in viral genome replication, while the potential involvement of lipid metabolism in later stages of the BVDV life cycle, including viral assembly, maturation, and release, requires further investigation. Second, the specific lipid species and metabolic pathways involved in fatty acid oxidation-mediated promotion of BVDV replication remain to be elucidated. In addition, although we identified the BVDV core protein as an important regulator of lipid droplet remodeling, whether other viral proteins, such as NS4B and NS5A, participate in lipid metabolic regulation or function synergistically with core protein, remains unknown. Future studies are needed to further clarify the complex interactions between BVDV proteins and host lipid metabolism.

## Conclusions

This study reveals that BVDV infection promotes intracellular accumulation of LDs, which are subsequently mobilized by the virus via lipolysis. By increasing contact between LDs and mitochondria, lipolysis-dependent FFAs are rapidly transferred to mitochondria for oxidation, thereby promoting viral replication. The BVDV core protein targets LDs and recruits FASN and ATGL, thereby promoting the mobilization of LDs. This core protein also interacts with mitochondria, potentially providing a bridge for the transfer of FAs from LDs to mitochondria. Our findings help elucidate the pathogenic mechanisms of BVDV infection and identify potential targets for therapies aimed at controlling BVDV transmission.

## Supplementary Information

**Additional file 1. BVDV promotes lipid droplet accumulation in OA-treated bovine cells.**

**Additional file 2. Effects of compound treatment on bovine cell viability.**

**Additional file 3. BVDV enhances the interaction between neutral lipase and LDs instead of increasing LD turnover in lysosomes.**

**Additional file 4. BVDV induces mitochondrial membrane depolarization in bovine cells.**

**Additional file 5. BVDV core protein associates with lipid droplets and interacts with TOMM20 under oleic acid-induced lipid accumulation.**

## Data Availability

The data will be shared upon request by the readers.

## Abbreviations

2-BP: 2-Bromopalmitate
AH: Amphipathic helix
ATGL: Adipose triglyceride lipase
BafA: Bafilomycin A1
BEECs: Bovine endometrial epithelial cells
BVDV: Bovine viral diarrhea virus
CPT: Carnitine palmitoyltransferase
DAG: Diacylglycerol
DGAT: Diacylglycerol acyltransferase
DNL: De novo lipogenesis
ER: Endoplasmic reticulum
FA: Fatty acid
FAO: Fatty acid oxidation
FASN: Fatty acid synthase
FFA: Free fatty acid
hpi: Hours post-infection
HSL: Hormone-sensitive lipase
LAL: Lysosomal acid lipase
LD: Lipid droplet
MCC: Manders’ colocalization coefficient
MCS: Membrane contact site
MDBK: Madin–Darby bovine kidney
MOI: Multiplicity of infection
mTORC1: Mechanistic target of rapamycin complex 1
OA: Oleic acid
PA: Palmitic acid
PCC: Pearson’s correlation coefficient
PI: Persistently infected
PLIN: Perilipin
RO: Replication organelle
TAG: Triacylglycerol
TC: Total cholesterol
