# Supplementary material for: Bovine viral diarrhea virus (BVDV) relies on cellular lipid droplet biogenesis and lipolysis to provide energy for viral replication
Source: Vet Res. 2026 Sep 19;57:168. doi: 10.1186/s13567-026-01834-7 (PMC13589865; doi:10.1186/s13567-026-01834-7)
Supplement: Supplementary file 1 [file 13567_2026_1834_MOESM1_ESM.doc]

**Additional file 1. BVDV promotes lipid droplet accumulation in OA-treated bovine cells.** BEEC cells were treated with 200 μM oleic acid (OA) for 12 h. BEECs were infected with BVDV strain AV67 at an MOI of 5 and fixed at 12 hpi. All cells were stained with BODIPY 493/503 to visualize LDs (green) and DAPI to visualize the cell nuclei (blue). Images are representative of three independent experiments. The scale bar represents 20 μm.
