# Supplementary material for: Bovine viral diarrhea virus (BVDV) relies on cellular lipid droplet biogenesis and lipolysis to provide energy for viral replication
Source: Vet Res. 2026 Sep 19;57:168. doi: 10.1186/s13567-026-01834-7 (PMC13589865; doi:10.1186/s13567-026-01834-7)
Supplement: Supplementary file 2 [file 13567_2026_1834_MOESM2_ESM.doc]

**Additional file 2. Effects of compound treatment on bovine cell viability.** Cell viability was assessed using a Cell Counting Kit-8 (CCK-8) assay after treatment with the indicated concentrations of the compounds for 36 h. Absorbance was measured at 450 nm. The effects of the compounds on cell viability are shown in additional file 2.
