# Supplementary material for: Bovine viral diarrhea virus (BVDV) relies on cellular lipid droplet biogenesis and lipolysis to provide energy for viral replication
Source: Vet Res. 2026 Sep 19;57:168. doi: 10.1186/s13567-026-01834-7 (PMC13589865; doi:10.1186/s13567-026-01834-7)
Supplement: Supplementary file 3 [file 13567_2026_1834_MOESM3_ESM.doc]

**Additional file 3. BVDV enhances the interaction between neutral lipase and LDs instead of increasing LD turnover in lysosomes.** (A) Mock or BVDV-infected (MOI = 5) MDBK cells were incubated in a maintenance medium. At 12 hpi, cells were fixed and stained with an antibody to HSL (red) and BODIPY 493/503 to visualize LDs (green). The nuclei were stained with DAPI (blue). The rightmost image shows the co-localized pixels as white. (B) Quantification of the colocalization of HSL and LDs by calculation of the PCC and MCC between signals in the red and green channels in experimental and control groups (more than 20 cells; n = 3 biological replicates).
