# Supplementary material for: Bovine viral diarrhea virus (BVDV) relies on cellular lipid droplet biogenesis and lipolysis to provide energy for viral replication
Source: Vet Res. 2026 Sep 19;57:168. doi: 10.1186/s13567-026-01834-7 (PMC13589865; doi:10.1186/s13567-026-01834-7)
Supplement: Supplementary file 4 [file 13567_2026_1834_MOESM4_ESM.doc]

**Additional file 4. BVDV induces mitochondrial membrane depolarization in bovine cells.** (A) Mock or BVDV-infected cells were stained with the mitochondrial membrane potential-sensitive dye JC-1 at 12 hpi. Red fluorescence represents JC-1 aggregates indicative of normal mitochondrial membrane potential, whereas green fluorescence represents JC-1 monomers indicative of mitochondrial depolarization. (B) Quantification of the fluorescence intensity ratio of JC-1 aggregates (red) to monomers (green) in mock- and BVDV-infected cells (n = 3 biological replicates).
