# Supplementary material for: Bovine viral diarrhea virus (BVDV) relies on cellular lipid droplet biogenesis and lipolysis to provide energy for viral replication
Source: Vet Res. 2026 Sep 19;57:168. doi: 10.1186/s13567-026-01834-7 (PMC13589865; doi:10.1186/s13567-026-01834-7)
Supplement: Supplementary file 5 [file 13567_2026_1834_MOESM5_ESM.doc]

**Additional file 5. BVDV Core protein associates with lipid droplets and interacts with TOMM20 under oleic acid-induced lipid accumulation.** (A) Schematic representation of the secondary structure of the BVDV Core protein, with α-helices and β-strands indicated in pink and yellow, respectively. (B) Predicted three-dimensional structure of the BVDV Core protein generated using the RoseTTAFold model. The α-helices (AH1 and AH2) are highlighted. (C) Localization of EGFP-Vec or EGFP-Core and lipid droplets in bovine cells under steady-state or oleic acid (OA)-treated conditions. Cells were stained with Nile red (red) to visualize lipid droplets and DAPI (blue) to label nuclei. (D) Colocalization of EGFP-Core or Core-EGFP with lipid droplets in OA-treated cells, as visualized by Nile red staining. (E) Effects of BVDV Core expression and OA treatment on autophagy-related proteins. The levels of LC3-I, LC3-II, and p62 were analyzed by Western blotting. GFP was used to confirm Core expression. (F) Cells were transfected with pEGFP-C1 or pEGFP-Core plasmid for 12 h and treated with 200 μM OA for another 12 h. At 36 h, protein interactions were detected by immunoprecipitation using anti-EGFP magnetic agarose beads and immunoblotting analysis with TOMM20 antibodies.
